# Disease Variants of FGFR3 Reveal Molecular Basis for the Recognition and Additional Roles for Cdc37 in Hsp90 Chaperone System

**DOI:** 10.1016/j.str.2018.01.016

**Published:** 2018-03-06

**Authors:** Tom D. Bunney, Alison J. Inglis, Domenico Sanfelice, Brendan Farrell, Christopher J. Kerr, Gary S. Thompson, Glenn R. Masson, Nethaji Thiyagarajan, Dmitri I. Svergun, Roger L. Williams, Alexander L. Breeze, Matilda Katan

**Affiliations:** 1 Institute of Structural and Molecular Biology, Division of Biosciences, University College London, Gower St, London WC1E 6BT, UK; 2 Medical Research Council (MRC) Laboratory of Molecular Biology, Cambridge CB2 0QH, UK; 3 Astbury Centre for Structural Molecular Biology, Faculty of Biological Sciences, Leeds LS2 9JT, UK; 4 European Molecular Biology Laboratory (EMBL) Hamburg Outstation, DESY, Hamburg, Germany

**Keywords:** Cdc37 cochaperone, Hsp90 chaperone, client kinases, fibroblast growth factor receptors, cancer, disease-linked mutations, structural and mechanistic insights, protein folding

## Abstract

Receptor tyrosine kinase FGFR3 is involved in many signaling networks and is frequently mutated in developmental disorders and cancer. The Hsp90/Cdc37 chaperone system is essential for function of normal and neoplastic cells. Here we uncover the mechanistic inter-relationships between these proteins by combining approaches including NMR, HDX-MS, and SAXS. We show that several disease-linked mutations convert FGFR3 to a stronger client, where the determinant underpinning client strength involves an allosteric network through the N-lobe and at the lobe interface. We determine the architecture of the client kinase/Cdc37 complex and demonstrate, together with site-specific information, that binding of Cdc37 to unrelated kinases induces a common, extensive conformational remodeling of the kinase N-lobe, beyond localized changes and interactions within the binary complex. As further shown for FGFR3, this processing by Cdc37 deactivates the kinase and presents it, in a specific orientation established in the complex, for direct recognition by Hsp90.

## Highlights

• Several disease-linked mutations convert FGFR3 to a stronger client of Hsp90/Cdc37
• Enhanced interaction with Cdc37 is underpinned by a weakened N-lobe network
• Cdc37 binding to unrelated kinases induces their common, extensive remodeling
• Kinase remodeling and the kinase/Cdc37 architecture allow recognition by Hsp90

## Graphical Abstract

**Figure:**
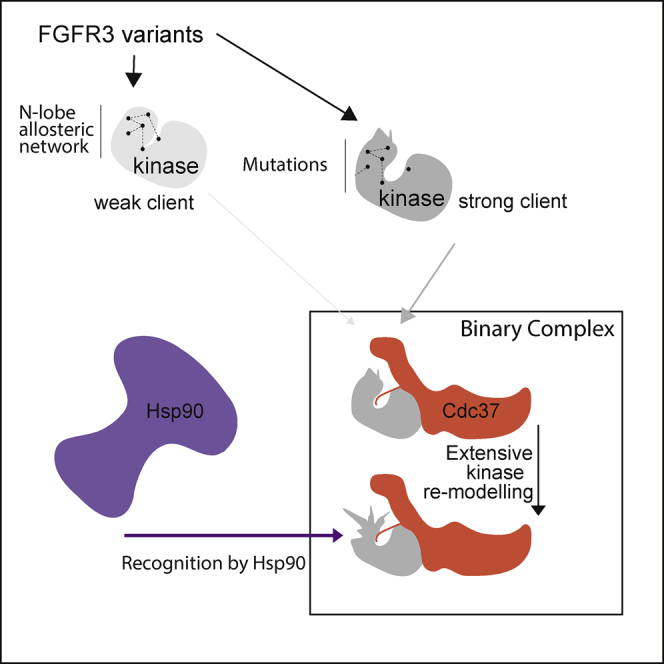

## Teaser

In their work, Bunney et al. propose a model in which the cochaperone Cdc37 plays a dual role in binding and remodeling its kinase clients, thus allowing recognition by the chaperone, Hsp90. The overall architecture of a Cdc37/FGFR3 complex is also presented.

## Introduction

Protein folding is a fundamental process guarded by the proteostasis network, including the Hsp90 family of chaperones, which facilitate the maturation of a wide range of client proteins. The Hsp90 clients are enriched in signal transduction components, such as protein kinases, which are crucial in regulating diverse cellular functions (Karagoz and Rudiger, 2015, Taipale et al., 2010). Hsp90 interacts with its clients thorough the involvement of different classes of cochaperones to exert marked effects on physiological and evolutionary processes as well as disease development; for example, interactions with protein kinases require kinase-specific cochaperone Cdc37 (Jarosz et al., 2010, Karagoz and Rudiger, 2015, Valastyan and Lindquist, 2014). In cases where proteins acquire pathological functions through mutation, such mutations often also destabilize protein structure. Hsp90 can rescue their folding or stability defects, thus enabling emergence of new or aberrant functions; among examples are Hsp90-dependent oncogenic variants with point-mutations, deletions or fusions, including kinases vSrc, HER2, BCR-ABL, B-Raf, and ELM4-ALK (Valastyan and Lindquist, 2014).

Among receptor tyrosine kinases, fibroblast growth factors receptors (FGFRs) have been linked to aberrant signaling in several developmental syndromes and a broad range of malignancies; this evidence has been accompanied by rapid progress in targeting FGFRs (Dieci et al., 2013, Katoh, 2016). It has also been reported that FGFRs, and in particular FGFR3, are clients for the Cdc37/Hsp90 chaperone machinery in various cellular systems and that the dependence on the chaperone is further enhanced for some cancer-associated FGFR gene fusion products (Acquaviva et al., 2014, Jin et al., 2011, Laederich et al., 2011, Taipale et al., 2012). It is, however, not clear how disease-linked point mutations in FGFR affect interactions with Cdc37 and Hsp90. Moreover, as for other client kinases, molecular features underpinning such interactions remain elusive.

Studies of the factors that govern the Cdc37/Hsp90 client status of protein kinases have failed to identify involvement of any common, specific kinase structural motifs. Rather, the key determinant appears to be global thermal stability, presumably related to the abundance and properties of their meta-stable folds (Boczek et al., 2015, Taipale et al., 2012, Taipale et al., 2013). It is well established that Cdc37 plays an obligate role in recognition and recruitment of kinases to Hsp90 as substrates (notably, client kinases are not recognized by Hsp90 in the absence of Cdc37) (Karagoz and Rudiger, 2015, Taipale et al., 2012). Structural and molecular mechanistic studies of binary and ternary interactions have mainly focused on two kinases, Cdk4 and B-Raf (Eckl et al., 2015, Keramisanou et al., 2016, Polier et al., 2013, Vaughan et al., 2006, Verba et al., 2016). Despite some detailed insights, it remains a challenge to generalize implications of these studies beyond the specific kinases exemplified. Furthermore, precise molecular features that determine or tune the level of kinase “clientness” remain undefined. Similarly, structural information for the kinase/Cdc37 complex is lacking, as is an understanding of the extent, sites, and consequences of conformational changes in kinases following binding to Cdc37.

Here we focus on FGFRs, a structurally and dynamically well-defined system, that has not been previously studied as a Cdc37/Hsp90 client in the mechanistic and structural contexts. Together with comparative observations for an established strong client, B-Raf, we address some of the key outstanding questions about client kinases by applying a range of methods, including nuclear magnetic resonance (NMR), hydrogen/deuterium exchange mass spectrometry (HDX-MS), and small-angle X-ray scattering (SAXS). After establishing that clinically relevant FGFR3 variants that become better clients are less-stable proteins, we highlight an allosteric network as a crucial determinant of kinase stability and consequently of the recognition by Cdc37. Furthermore, we describe multiple sites of interaction and the overall architecture of the FGFR3 kinase/Cdc37 complex. Importantly, we also establish the consequences of Cdc37 binding and show very extensive remodeling of FGFR3 kinase and similar changes in B-Raf. We propose that, in the kinase/Cdc37 complex, the remodeled regions of the partially inactivated kinase are accessible for the recognition by Hsp90, ultimately resulting in protection and refolding.

## Results

### Differences between FGFR1-4 and FGFR3 Disease-Linked Variants in the Ability to Form Complexes with Cdc37 and Hsp90

**Figure 1:**
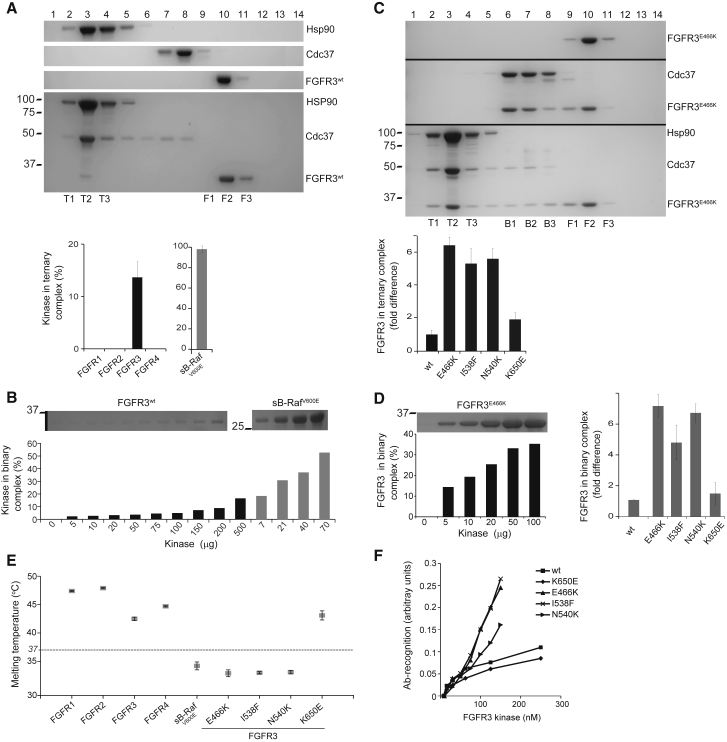
Interaction of FGFR Kinase Domains and FGFR3 Variants with Cdc37 and Hsp90 (A) SEC of isolated components Hsp90, Cdc37, and FGFR3^WT^ (top panels) and of their mixture (ratio 2:1:1), showing the ternary complex (fractions T1-T3) and free FGFR3^WT^ (fractions F1-F3) (bottom panel). The graphs (below) show quantitation of the incorporation of kinases in the ternary complex for FGFR1, 2, 3, and 4 (left), and for sB-Raf^V600E^ (right). The error bars represent the SD (n = 3). (B) Comparison of FGFR3^WT^ with sB-Raf^V600E^ using a pull-down binding assay using immobilized Cdc37. A quantification of the amount of kinase bound is shown in the lower panel. (C) SEC of FGFR3^E466K^ (top panel). SEC of a 1:1 mixture with Cdc37 shows the binary complex (fraction B1-B3) and free kinase (fractions F1-F3) (middle panel) and of a mixture with both Hsp90 and Cdc37 (ratio 2:1:1) showing the ternary complex (fraction T1-T3), a small amount of the binary complex (fractions B1-B3) and free kinase (fractions F1-F3) (bottom panel). The graph (below) shows quantification of FGFR3 in the ternary complex for the FGFR3^WT^, FGFR3^E466K^, FGFR3^I538F^, FGFR3^N540K^, and FGFR3^K650E^. The error bars represent the SD (n = 3). (D) Binding of FGFR3^E466K^ to immobilized Cdc37 (left) and comparison of binding of FGFR3^WT^ with FGFR3^E466K^, FGFR3^I538F^, FGFR3^N540K^, and FGFR3^K650E^ in this pull-down assay (right). The error bars represent the SD (n = 3). (E) Comparison of melting temperatures of kinase domains of FGFR1-4, sB-Raf^V600E^, and FGFR3^E466K^, FGFR3^I538F^, FGFR3^N540K^, and FGFR3^K650E^. The error bars represent the SD (n = 4). (F) Recognition of FGFR3^WT^, FGFR3^E466K^, FGFR3^I538F^, FGFR3^N540K^, and FGFR3^K650E^ by a polyclonal antibody prepared against a peptide corresponding to FGFR3 amino acid residues 467–495 (LS-C214248, Lifespan BioSciences). See also Figures S1–S4.

To further characterize FGFR/Cdc37/Hsp90 complexes identified in cellular settings (Laederich et al., 2011, Taipale et al., 2012), we performed reconstitution from purified components to first show that ternary and binary complexes with wild-type (WT) FGFR3 kinase domain (henceforth denoted FGFR3^WT^) can be assembled *in vitro*. The complexes were analyzed by size-exclusion chromatography (SEC) and pull-down experiments (Figures 1 and S1). We quantified the amount of FGFR3^WT^ incorporated in the ternary complex under defined conditions (ratio FGFR3^WT^:pCdc37:Hsp90, 1:1:2) and found that 12%–17% was present in the complex. Under the same conditions, we have not observed any FGFR1, 2, or 4 kinase domain in ternary complexes. We also compared the unrelated Ser/Thr B-Raf kinase domain, a well-established “strong” Cdc37 client, using a variant incorporating the V600E mutation as well as a number of other changes to ensure solubility (designated as sB-Raf^V600E^ [Tsai et al., 2008]), which, as expected, demonstrated higher occupancy for both ternary and binary complexes than FGFR3^WT^ (Figures 1A, 1B, and S1).

We further extended this *in vitro* analysis to a panel of over 20 cancer mutations in the kinase domain of FGFR3 some of which overlap with mutations found in developmental disorders. Recently, our analysis of this panel identified a number of mutations that increase non-stimulated kinase activity (including N540K and K650E substitutions) among many other (mostly infrequent) mutations that have no such effect (Patani et al., 2016). Using the same panel, we tested the ability of these variants of FGFR3 kinase domain (hereafter denoted FGFR3^N540K^, FGFR3^K650E^, etc.) to form binary and ternary complexes, using SEC and a pull-down assay (Figures 1C, 1D, S1E, and S2A). We identified three variants, FGFR3^E466K^, FGFR3^I538F^, and FGFR3^N540K^, which show higher occupancies in complexes compared with the WT or the K650E hotspot mutation (Figures 1C and 1D). Consistent with this, we measured *K*_D_ values for binding of Cdc37 to FGFR3^I538F^ and to FGFR3^WT^ using isothermal titration calorimetry (ITC), and found that Cdc37 bound tightly to FGFR3^I538F^ with a *K*_D_ of 1.8 μM, while binding to the WT variant was too weak to be detectable by ITC, indicating a *K*_D_ > mM (Figure S1F). Based on the comparative SEC and pull-down data (Figures 1C, 1D, and S1E) it is expected that the *K*_D_ for FGFR3^E466K^ and FGFR3^N540K^ would be within the range of 1–2 μM. Although these values are higher than the previously reported *K*_D_ for a variant of B-Raf (∼0.2 μM) (Polier et al., 2013), for a relative comparison of FGFR3 kinases we refer to FGFR3^WT^ as a “weak” client, and to FGFR3^E466K^, FGFR3^I538F^, and FGFR3^N540K^ as “strong” clients.

Among the variants that show a marked increase in incorporation in both binary and ternary complexes, FGFR3^E466K^, FGFR3^I538F^, and FGFR3^N540K^ (Figures 1C and 1D), only in the case of FGFR3^N540K^ is kinase activity greatly enhanced (FGFR3^E466K^ is similar to FGFR3^WT^ and FGFR3^I538F^ has slightly reduced kinase activity) (Patani et al., 2016). To test a possible correlation with protein stability, we further performed an analysis of kinase domain thermal stability for the panel of cancer mutations, WT FGFR1-4, and sB-Raf^V600E^. As shown in Figure 1E, FGFR3^WT^ is less stable compared with FGFR1, 2, and 4, while the stability of FGFR3^E466K^, FGFR3^I538F^, and FGFR3^N540K^ is further compromised to a level comparable with the thermal stability of the sB-Raf^V600E^ variant. In the case of the thermo-labile FGFR3 variants, recognition by an anti-FGFR3 antibody using ELISA was also enhanced. The highly active FGFR3^K650E^ had similar thermal stability and antibody recognition as the FGFR3^WT^ (Figures 1E and 1F). All other FGFR3 variants had stability and Cdc37 binding similar to the FGFR3^WT^ (Figure S2). Thus, using this comprehensive panel, we found that, while the activity among tested kinases differs considerably irrespective of the strength of Cdc37 binding, the observed decrease in thermal stability correlates well with enhanced client status. Furthermore, we also established that binding of FGFR3 inhibitors that reduce formation of binary and ternary complexes, correlates with the increase in thermal stability of the inhibitor-bound kinase (Figures S3A–S3C). Differences in antibody recognition (Figure 1F) provide further supporting evidence that distinct molecular properties, which do not correlate with the kinase activity, are specifically associated with stronger clients.

Using the FGFR3^E466K^ variant, we also obtained some further information of broader relevance for kinase/Cdc37 interactions. Consistent with previous observations for other kinases (Polier et al., 2013, Oberoi et al., 2016), we have found that phosphorylation of Cdc37 (on S13 by CK2) has no effect on the binary complex, but enhances formation or stability of the ternary complex (Figures S4A and S4B). However, in contrast to a previous report (Liu and Landgraf, 2015), our data suggest that the change in Cdc37 phosphorylation status is not linked to a conformational change in this protein (Figure S4C).

### Characterization of Weak versus Strong Client Kinases

**Figure 2:**
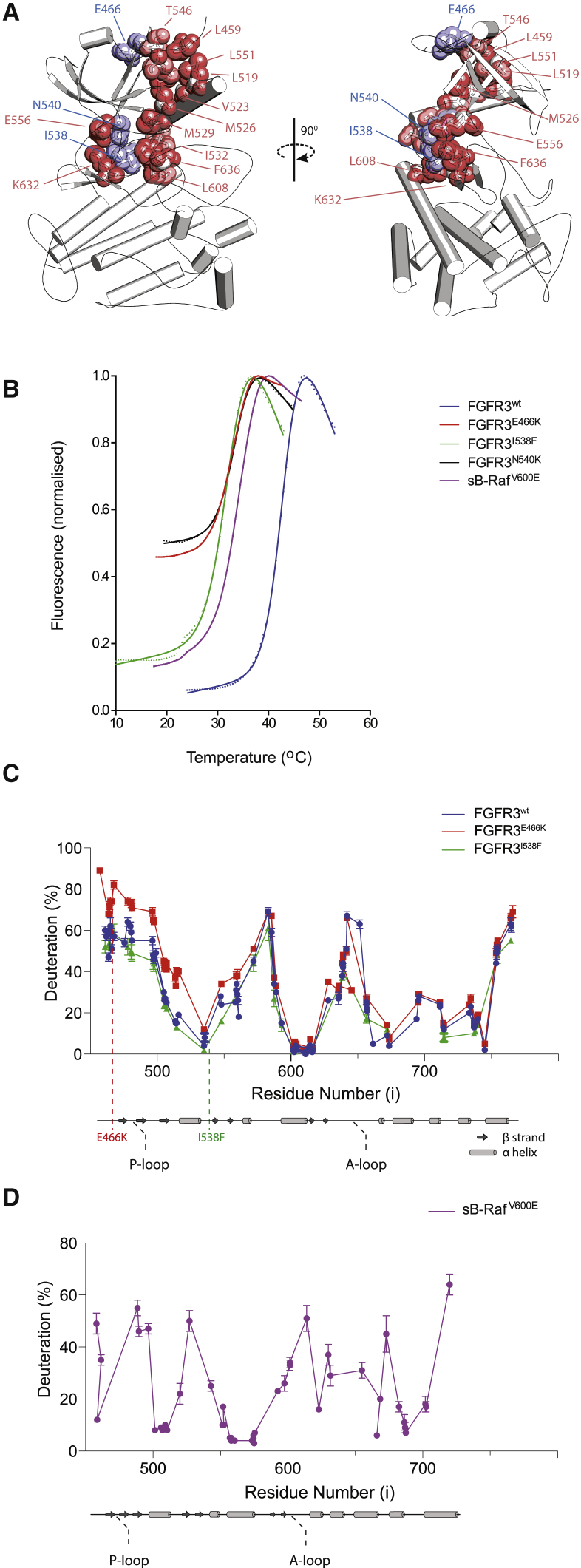
Comparison of Overall Structural Differences between Client Kinase Domains (A) The FGFR allosteric network running through the N-lobe and linking to the activation loop and the lobe interface. Cartoon representation of an FGFR3^WT^ model, with amino acid residues corresponding to sites of disease-linked mutations found to destabilize the kinase domain colored blue and all other residues within the network shown as pink. (B) Normalized thermal-shift curves for the indicated kinase domain variants. (C) A global deuteration profile of FGFR3 proteins. A linear plot of deuteration levels across FGFR3^WT^ (blue) and two variants FGFR3^E466K^ (red) and FGFR3^I538F^ (green). The percentage of deuteration at the 0.3 s time point for each peptide is plotted against the peptide mid-point (i); secondary structure and key features of the kinase domain region are also shown and mutations indicated by dotted lines (bottom). The data plotted are means ± SD (n = 3). The error bars smaller than the icon are omitted. (D) A global deuteration profile for the kinase domain of sB-Raf^V600E^; the plot (top) and secondary structure (bottom) are represented as in (C).

Destabilizing mutations in FGFR3 kinase domain are located within the N-terminal part of the N-lobe (E466K) and within features described as the “DFG latch” (I538F) and “molecular brake” (N540K), previously linked to N-lobe rotation and activation mechanisms (Chen et al., 2017). Based on our further analysis of the available data (including over 30 crystal structures of FGFR kinase domains), all three mutated residues are likely to be connected within a larger allosteric network (Figure 2A). In addition to local changes, perturbations in this network are expected to have, at least to some degree, an effect on the overall structural integrity of the N-lobe and the lobe interface. To test this possibility, our further experiments aimed to provide direct structural information.

Detailed comparison of FGFR3^E466K^, FGFR3^I538F^, and FGFR3^N540K^ reveals some differences in thermal-shift curves between these less stable variants; curves for FGFR3^E466K^ and FGFR3^N540K^ exhibit greater basal fluorescence (suggesting greater hydrophobic exposure) even at lower temperatures (20°C) (Figure 2B). Also, expression of FGFR3^E466K^ and FGFR3^N540K^ appears to be more impaired (expression levels WT > I538F ≫ E466K > N540K), and amounts of FGFR3^N540K^ are insufficient for more extensive characterization. Therefore, to further assess the impact of mutations on the structure, we performed HDX-MS using only FGFR3^WT^, FGFR3^I538F^, and FGFR3^E466K^ and NMR spectroscopy using the two most highly expressed proteins, FGFR3^WT^ and FGFR3^I538F^.

For HDX-MS, we compared the extent of deuteration in FGFR3^WT^, FGFR3^I538F^, and FGFR3^E466K^ with that in sB-Raf^V600E^ (Figures 2C and 2D). For all proteins, there are some common features that are characterized by very low (αC and αE helices) or very high (N and C termini, A loop, and regions of β strands) rates of solvent exchange. Interestingly, among FGFR3 kinase domains, the less stable FGFR3^E466K^ has a higher percentage of deuteration in peptides derived from across the entire N-lobe (Figure 2C). This is consistent with the key position of E466 residue within the N-lobe allosteric network encompassing the N terminus (including L459), β4-β5 linker (T546 and L551), and αC helix (L519, V523 and M526), which is further connected to other elements of the network (Figure 2A). In contrast, HDX changes in I538F are less significant and similar to the WT, suggesting a subtler effect that is undetectable by HDX-MS, but nevertheless sufficient to considerably enhance binding to Cdc37, as corroborated by our ITC data (Figure S1F).

**Figure 3:**
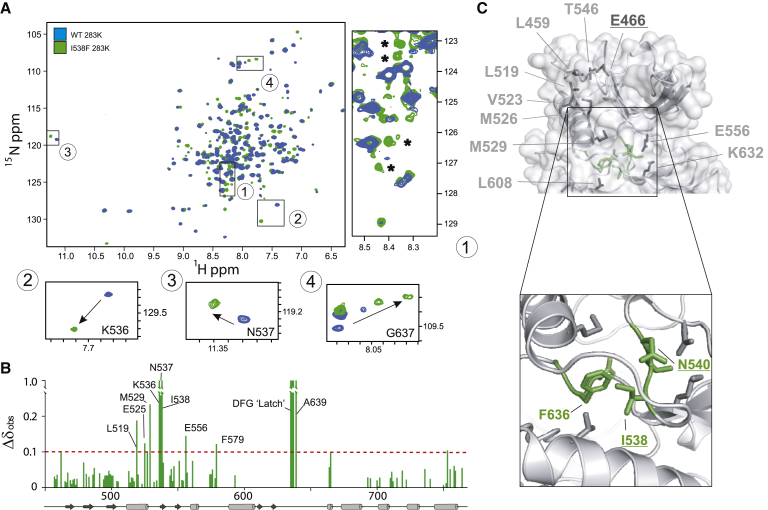
Comparison of FGFR3 Variants by NMR (A) Superimposed 2D amide TROSY-HSQC experiments for FGFR3^WT^ (blue) and FGFR3^I538F^ mutant (green). Comparison was performed at 283K. Highlighted in four boxes (1–4) are the most prominent differences; new sharp peaks in box 1 are indicated by (^∗^). (B) NMR changes were quantified by CSP analysis. The histogram shows in green the successfully assigned residues transferred from WT at 298K. (C) FGFR3 residues (shown as side chains and labeled) involved in the allosteric network encompassing the N-lobe and the N-lobe/C-lobe interface are highlighted in a cartoon and surface representation of the kinase. The enlarged N-lobe/C-lobe interface is shown in the inset below with F638 (from the DFG motif), I538 (part of the “DFG latch”), and N540 (from the “molecular brake”) highlighted. Underlined residues (E466 within the N-terminal part of the N-lobe and I538 and N540 at the interface), corresponding to mutations that destabilize FGFR3 kinase domain, are shown as a part of an extensive, interconnected allosteric network highlighted by NMR changes. See also Figure S5.

To gain more detailed insights into differences between the FGFR3^WT^ and FGFR3^I538F^ that represent weak and strong clients, respectively, we compared them using NMR spectroscopy. We have previously applied NMR to analyze FGFR3^WT^ (Bunney et al., 2015, Patani et al., 2016), aided by our full NMR assignment of FGFR1 (Klein et al., 2015, Vajpai et al., 2014). Here, using a combination of selective labeling and 3D experiments, we were able to achieve a backbone assignment for FGFR3^WT^ of 75%. A direct comparison of the 2D ^1^H-^15^N TROSY-HSQC spectra of FGFR3^WT^ and the less stable FGFR3^I538F^ variant was possible by reducing the temperature to 283K (in these conditions FGFR3^I538F^ samples were stable long enough to acquire 2D spectra) (Figure S5). Overall, spectra of FGFR3^WT^ and FGFR3^I538F^ are similar (Figure 3A), confirming that the global structure of the two variants is conserved. As expected the aromatic ring current arising from the I538F mutation has a strong local effect on the chemical shifts of the neighboring amino acid residues K536 and N537 (Figure 3A, inserts 2 and 3). There are also further effects on the “DFG latch,” including disappearance of F636 and D635 and major shifts of G637 and A639 in the A-loop region (Figure 3A, inserts 1 and 4). We noted significant chemical shift perturbations (CSPs) for several amino acid residues within the allosteric network (E556, K536, I532, M529, and E525) (Figures 3B and 2A), which might result in reduced overall stability of the N-lobe and its interface with the C-lobe. Strikingly, in the spectrum of FGFR3^I538F^ we observed the appearance of new, sharp peaks in the random-coil chemical shift region that are not present in FGFR3^WT^ (Figure 3A, insert 1). We interpret these peaks as signaling localized unfolding that arises from perturbation of the extended allosteric network due to the I538F substitution, and that likely contributes to the reduced thermal stability of this variant. As summarized in Figure 3C, NMR data highlight changes consistent with the proposed allosteric connectivity, where the I538F mutation results not only in changes in neighboring residues, but also in decreases in overall stability and at the interface between the lobes. We have also noted that the binding of an FGFR inhibitor, PD173074, which increases kinase domain thermal stability was accompanied by prominent CSPs in the N-lobe allosteric network of FGFR3^WT^ (Figures S3D and S3E). With respect to localized unfolding, the random-coil peaks, already present in free FGFR3^I538F^ (but absent from FGFR3^WT^) (Figure 3A, insert 1) correlate remarkably to those observed to intensify upon Cdc37 complex formation (see next section and Figure 5).

### Molecular Changes in Kinases Following Cdc37 Binding

**Figure 4:**
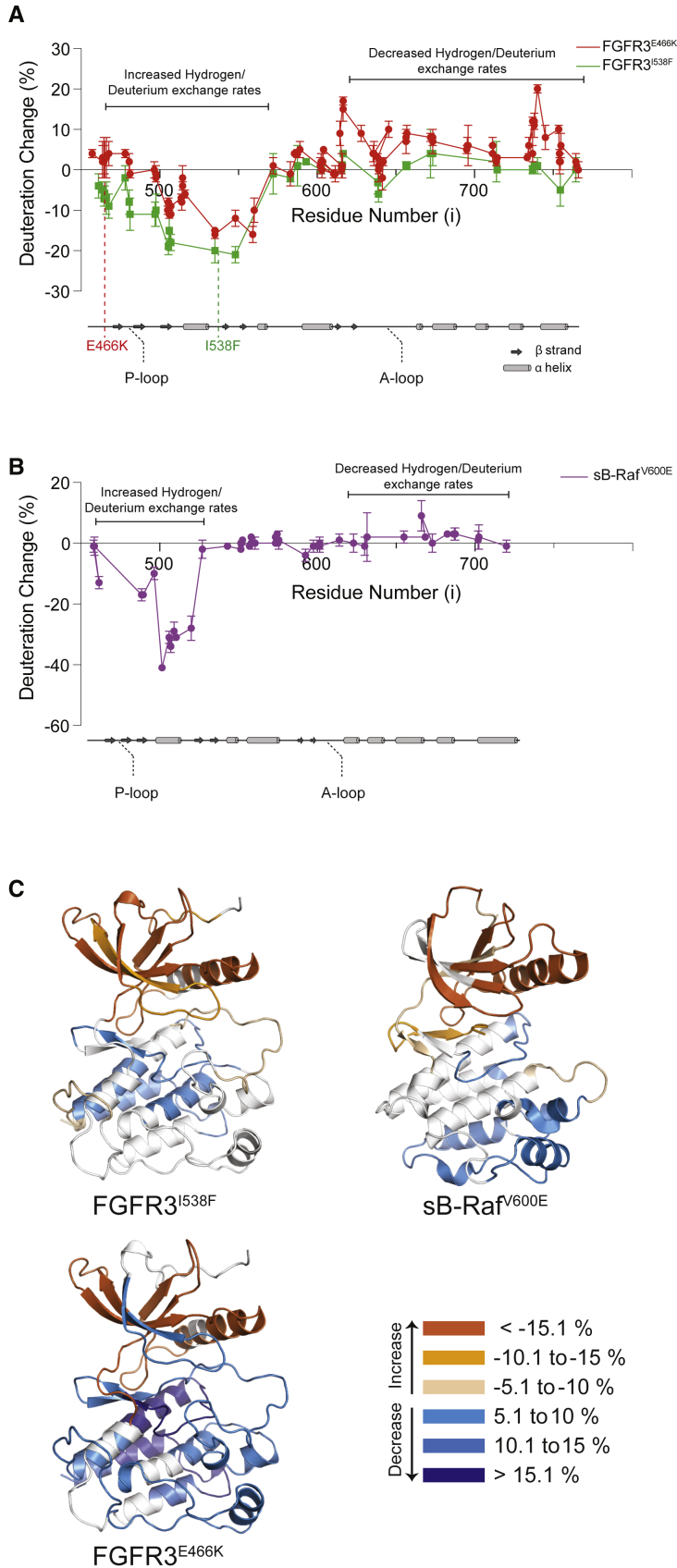
Global Changes in Kinase Domains Following Interaction with Cdc37 (A) The difference in HDX for FGFR3 variants in their free form and when in complex with Cdc37. Percent deuteration change was calculated by subtracting the deuteration of each peptide of FGFR3 complexed with Cdc37 from the same peptide of free FGFR3 at the 0.3 s time point. The data plotted are means ± SD (n = 3). (B) The same depiction as in (A) for sB-Raf^V600E^. (C) Ribbon representations of FGFR3 and sB-Raf^V600E^ kinase domains with HDX differences mapped onto them. Peptides with significant changes (<5%) upon addition of excess Cdc37 have been colored according to the key. For each peptide, the time point with the largest difference between the free and bound states was used in this analysis. See also Figure S6 and Table S1.

To identify changes in the kinase architecture that result from the binding of Cdc37, FGFR3^WT^, FGFR3^I538F^, and FGFR3^E466K^ were analyzed by HDX-MS following the addition of a stoichiometric excess of Cdc37 (Figures 4 and S6; Table S1). For comparison with an established strong client, we again included sB-Raf^V600E^ in the analysis. Using this method, the consequences of the weak binding to FGFR3^WT^ were barely detectable. However, binding of Cdc37 to all other kinase variants had a pronounced effect that, in each case, showed remarkable similarities. Importantly, a significant portion of the N-lobe shows an increase in solvent exchange rate while several regions of the C-lobe were observed to decrease their exchange rate upon binding (Figures 4A and 4B). Mapping of the peptides which showed differences in solvent exchange rates on to kinase structures highlighted αC and the majority of the N-lobe β strands as regions that had dramatic increases of their solvent exchange rates upon the addition of Cdc37 in all kinases (Figure 4C). The regions that show a reduction in their solvent exchange rates in the C-lobe are most extensive for the most labile FGFR3^E466K^ variant, and overlap with those of FGFR3^I538F^ and sB-Raf^V600E^. These regions include FGFR3 amino acid residues 592–602, 611–628, 670–684, and 732–752; in sB-Raf^V600E^ the corresponding protected residues are 568–581, 619–627, and 659–707.

HDX-MS does not afford a direct map of protein-protein interaction sites. Nevertheless, regions that experience a decrease in exchange with solvent upon addition of a binding partner have a high probability of involvement in protein-protein interfaces (Konermann et al., 2011). HDX-MS supports very substantial conformational changes in client kinases upon Cdc37 binding that, in addition to direct interactions, result in a less-structured N-lobe.

**Figure 5:**
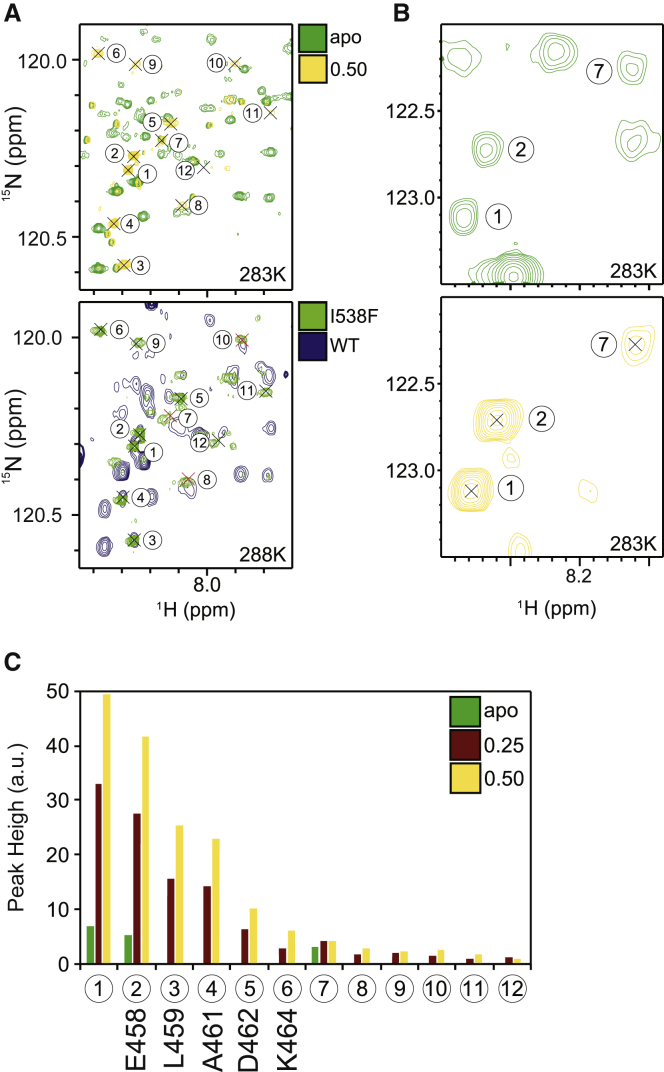
Changes in FGFR3 Following the Interaction with Cdc37 Assessed by NMR (A) Selected region of ^1^H-^15^N TROSY-HSQC spectra of FGFR3^I538F^ in the absence and presence of 0.5 equivalents of Cdc37, acquired at 283K (top subpanel); for comparison, spectra of FGFR3^WT^ (navy) and FGFR3^I538F^ (lime), both in the presence of 0.7 equivalents of Cdc37 and acquired at 288K are also shown (bottom subpanel). Coloring of the top subpanel is as in panel (A) The location of sharp peaks (1–12, numbered according to relative intensity) is indicated in both panels and red crosses show approximate locations. (B) Selected spectral regions of FGFR3^I538F^ in the absence (top subpanel) and presence (bottom subpanel) of 0.5 equivalents of Cdc37, illustrating the presence of sharp peaks 1, 2, and 7 in the free state and their increased intensity in the complex. (C) Peak heights of sharp peaks 1–12 in FGFR3^I538F^ in the absence (green) and presence of 0.25 (maroon) and 0.5 (yellow) equivalents of Cdc37. Assignments of these peaks are given below their respective peak number.

To complement the insights from HDX-MS, we again employed 2D HSQC NMR and performed titration experiments in which unlabeled Cdc37 (at molar ratios of 0.25 and 0.5) was added to ^15^N-labeled samples of FGFR3^I538F^ (Figure 4). Because of the low-μM *K*_D_ and 1:1 stoichiometry for binding of Cdc37, as determined by ITC (Figure S1F), we anticipated that resonances of the unbound kinase should still be present in these spectra at ∼75% and ∼50%, respectively, of their intensity in the absence of Cdc37, and that any new resonances arising from the bound conformation(s) of FGFR3^I538F^ should be significantly broadened as a result of the large size (82 kDa) of the complex. Surprisingly, we found that in the presence of Cdc37, FGFR3^I538F^ resonances were much more extensively reduced in intensity than suggested by the stoichiometric ratios employed; this implies that exchange between free and Cdc37-bound conformation(s) of the kinase occurs on an intermediate (microsecond to millisecond) timescale in the complex, even for this relatively high affinity interaction. Another striking observation (in the context of the overall resonance broadening) was the presence of a number of intense, sharp peaks in the random-coil region of the spectrum (Figures 5A and 5B). Using a combination of 3D experiments and selective labeling, we were able to assign these amide resonances to the N-terminal region of the FGFR3 N-lobe (Figure 5C). Although some of the sharp peaks were already present in the free state and observed as a distinct feature of FGFR3^I538F^ (as highlighted in Figure 3A), they intensify notably upon addition of Cdc37, implying that this region becomes less folded and more mobile in the complex with the cochaperone. Interestingly, titration of FGFR3^WT^ (which binds only weakly to Cdc37, as determined by ITC [Figure S1F]) with higher concentrations of the cochaperone also resulted in appearance of these sharp peaks at identical chemical shifts, despite their complete absence, as noted above, from the spectrum of free FGFR3^WT^ (Figure 5A). Our NMR results are thus consistent with our observations from HDX-MS that Cdc37 causes more extensive remodeling of the kinase N-lobe (Figure 4); however, further interpretation of global changes to the kinase by NMR are currently elusive because of the extent of conformational exchange broadening in the large complex. Further analysis using circular dichroism (CD) to compare FGFR3^I538F^ alone and in complex with Cdc37 have shown that global secondary structure composition is largely preserved (<15% change). Taken together, our data from HDX-MS, NMR, and CD analyses support a substantial remodeling of the kinase N-lobe tertiary structure with transient, localized unfolding, but without large perturbations within the secondary structure elements.

### Molecular Changes in Cdc37 Following Kinase Binding and Insights into a Kinase/Cdc37 Complex

**Figure 6:**
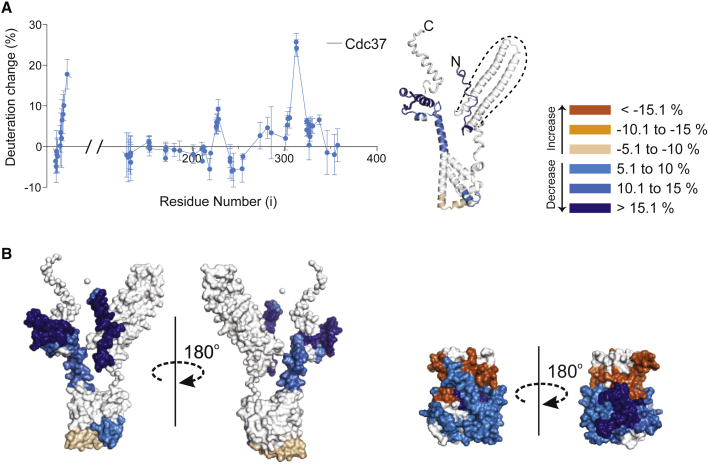
Changes in Cdc37 Following FGFR3 Kinase Binding and a Structural Model for the Cdc37/FGFR3 Kinase Domain Binary Complex (A) The difference in HDX for Cdc37 in its free form and when in complex with FGFR3^E466K^. For the regions covered (residues 29–119 are lacking in the analysis), percent deuteration change was calculated by subtracting the deuteration of each peptide of Cdc37 complexed with FGFR3 from the same peptide of free Cdc37 at the 0.3 s time point (left panel). Data plotted are the means ± SD (n = 3). Ribbon representations of a Cdc37 model (based on structures and SAXS data-SASDBP9) with HDX differences mapped onto them. Peptides with significant changes (<5%) upon addition of excess FGFR3 have been colored according to the key. For each peptide, the time point with the largest difference between the free and bound states was used in this analysis (right panels). Region of Cdc37 outlined with a dotted line did not yield any data from the HDX-MS analysis. (B) Surface representations of Cdc37 and FGFR3 kinase domain (depicted at the same scale) with HDX differences between the free and complex forms mapped and colored according to the key in (A).

To analyze molecular changes in Cdc37 resulting from kinase binding, we used HDX-MS and focused on the FGFR3^E466K^ variant. As shown in Figure 6A, this analysis did not cover the entire Cdc37 sequence due to incomplete proteolysis in parts of the N-terminal sequence. Nevertheless, we identified two regions of Cdc37 that experience decreased HDX: the extreme N terminus (residues 5–28), and the C-terminal α helices (residues 302–341). Interestingly, the same regions have recently been identified in NMR experiments in which Cdc37 was titrated with sB-Raf^V600E^ (Keramisanou et al., 2016). Furthermore, most of the region of Cdc37 that was not covered by HDX-MS in our study, appears not to be involved in kinase interaction based on NMR (residues 40–105) (Keramisanou et al., 2016). In addition to changes in the N- and C-terminal regions, some smaller changes in HDX rate were observed by HDX-MS (middle region, residues 219–251 and 270–293; Figures 6A and 6B). Interestingly, changes in this middle region of Cdc37 were previously observed by NMR and attributed to conformational changes on complex formation, rather than direct involvement in a binding interface (Keramisanou et al., 2016).

**Figure 7:**
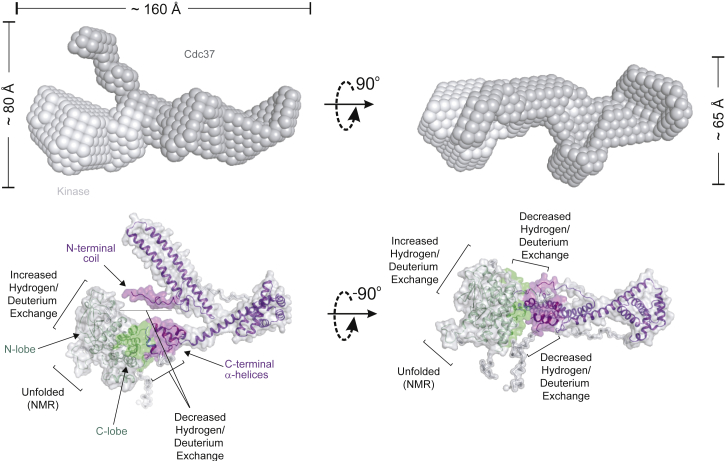
A Structural Model for the Cdc37/FGFR3 Kinase Domain Binary Complex SAXS envelope for the Cdc37/FGFR3 complex, with Cdc37 in dark and FGFR3 in light gray (top). The best fit for the complex is shown below as cartoon and surface representations of the kinase domain (green) and Cdc37 (purple). Notable features are labeled and regions identified as protected by HDX-MS shaded in corresponding colors. See also Figure S7 and Table S2.

In contrast to client kinases, which undergo very substantial changes in solvent exchange rate across the entire structure (Figure 4), changes in Cdc37 appear to be more localized and are mainly reflected in decreased solvent exchange rates (Figure 6). The insights from our HDX-MS analysis (different kinases show similar changes [Figure 4]; Cdc37 complexed to the FGFR3^E466K^ variant [Figure 6] shows HDX decreases in the regions previously identified by NMR in sB-Raf^V600E^ complexes [Keramisanou et al., 2016]) suggest sites that could be common across different kinase-Cdc37 interactions. However, this knowledge is not sufficient to define the spatial arrangement of Cdc37 and kinase within a binary complex. A complex of Cdc37 and FGFR3^I538F^ was therefore further analyzed by synchrotron SAXS (Svergun et al., 2013) to obtain low-resolution insights into the overall architecture of the complex (Figures 7 and S7; Table S2). Scattering curves were recorded for the single components and for the complex, and all three SAXS curves analyzed together to provide consistent models (Figure S7). The parameters of the analysis and the calculated models for both the individual components and the complex are summarized in Table S2. Although showing variations at higher resolution, the models derived from SAXS using both *ab initio* and hybrid rigid body approaches converge and predict highly asymmetric structures with distinct features. Notably, one lobe of the models is consistently occupied by the paired helices from Cdc37 (residues 29–119), while FGFR3^I538F^ can be fitted to a second lobe of the envelope, facing the terminal regions of Cdc37. The model in Figure 7 shows the best fit that is consistent with the measured scattering curves. An arrangement for the two components reveals that N- and C-terminal regions of Cdc37 interact mostly with the C-lobe of the kinase, with the N terminus extending further toward the N-lobe. As discussed further (see Discussion) this model, suggesting a multi-site nature of Cdc37 binding to client kinases, is also consistent with previous biochemical studies (Eckl et al., 2015, Keramisanou et al., 2016). We also obtained a model for uncomplexed Cdc37 using SAXS that suggests that the C- and N-terminal parts are in close proximity (Figures 7 and S7); this model assembles previously determined structures for the protein lacking the N-terminal region (127–378) (Roe et al., 2004) and a structure of the isolated N terminus (1–126) (Keramisanou et al., 2016).

**Figure 8:**
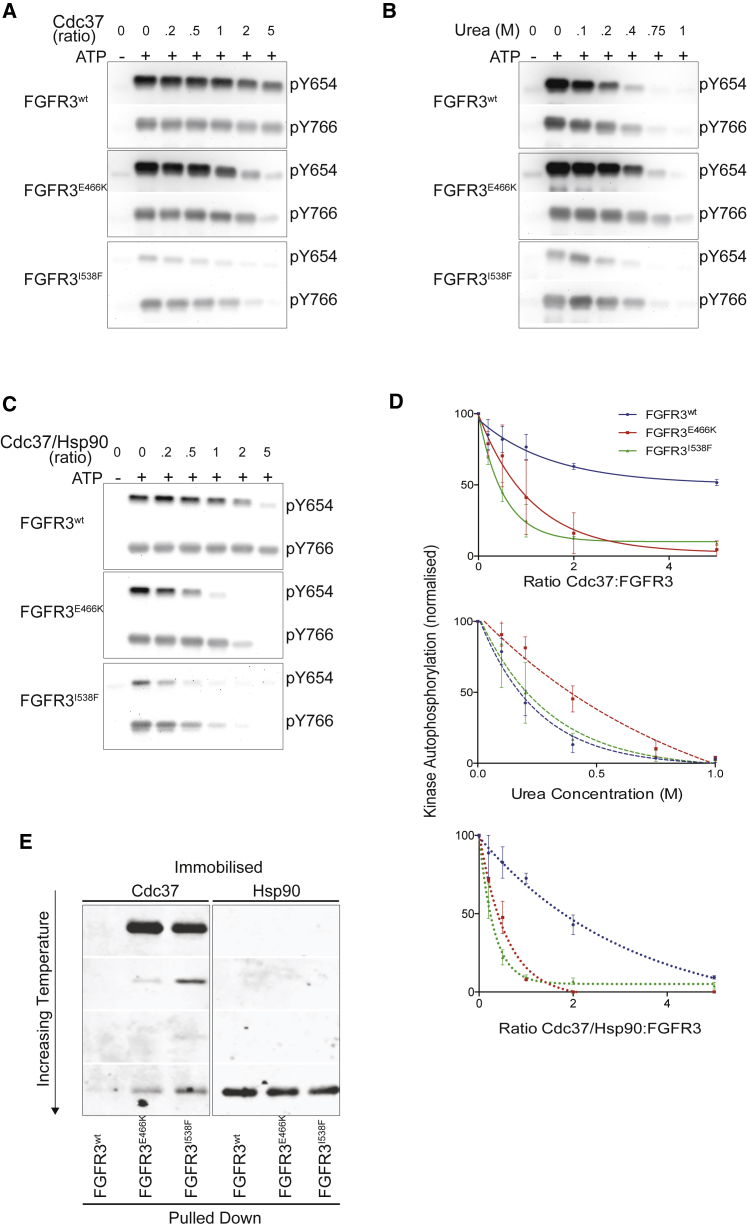
Functional Consequences of Cdc37 Binding to FGFR3 Kinases (A) Western blot utilizing antibodies that recognize phosphorylated tyrosine residues on FGFR3. Increasing ratios of Cdc37 to kinase are monitored for the effect on FGFR3^WT^, FGFR3^E466K^, and FGFR3^I538F^ auto-phosphorylation. (B) As (A) but the effect of increasing urea concentration is monitored for its effect on auto-phosphorylation of FGFR3 variants. (C) As (A) but the effect of increasing Hsp90/Cdc37 to kinase ratio is monitored for its effect on auto-phosphorylation of FGFR3 variants. (D) Decrease in normalized kinase auto-phosphorylation (pY654), following addition of Cdc37 (top), urea (middle), or Cdc37 together with Hsp90 (bottom). Data plotted are the means ± SD (n = 3). (E) Temperature dependence of client kinase binding to Cdc37 and Hsp90. Immobilized Cdc37 or Hsp90 are utilized to pull down FGFR3 variants at various temperatures (4°C, 15°C, 25°C, and 40°C). Bound protein is visualized using western blotting with a FGFR3 antibody.

The key insights from our structural studies imply that differences between weak and strong client kinases are relatively subtle, but that the interaction with Cdc37 results in substantial changes, most strikingly an increase in disorder of the N-lobe. To further substantiate these findings, we performed additional experiments to monitor how the interaction with Cdc37 affects kinase activity and how exposure of kinases to different temperatures affects protein-protein interactions (Figure 8). The interaction with Cdc37 results in an inhibition of kinase activity, with the FGFR3 variants with stronger binding to Ccd37 showing a more pronounced inhibition of auto-phosphorylation (Figures 8A and 8D). In particular, the inhibition of FGFR3^E466K^ by an equimolar concentration of Cdc37 is equivalent to exposure to 0.4 M urea (Figures 8B and 8D). However, unlike Cdc37, the effects of urea on all FGFR3 variants is similar (irrespective of their thermal stability differences), highlighting the specificity of Cdc37-induced unfolding compared with that of urea. The reduction of kinase activity by Cdc37 is even more marked when Hsp90 is included in the experiment (Figures 8C and 8D).

It has been documented that Hsp90 cannot recognize kinases in the absence of Cdc37 (Taipale et al., 2012). To test this in our system, different FGFR3 variants were incubated at increasing temperatures before being subjected to a pull-down on either immobilized Cdc37 or Hsp90 (Figure 8E). Although increasing temperature presumably induced structural instability, we noted reduced binding to Cdc37. However, at the highest temperature tested (40°C), direct binding of the kinase to Hsp90 was observed. This is consistent with the notion that recognition of clients by Cdc37 requires a high degree of structural preservation of the kinase fold, whereas recognition by Hsp90 relies on partially folded (or unfolded) structures.

## Discussion

We have focused on FGFR kinases, and here describe molecular determinants that influence recognition by Cdc37, the architecture of the kinase/Cdc37 complex, the extensive remodeling of kinase structure, and its mechanistic and functional consequences beyond binary complex formation, common to different client kinases.

FGFR kinases, and in particular FGFR3, have been recognized as clients of the Cdc37/Hsp90 system in cells (Laederich et al., 2011, Taipale et al., 2012), and here we demonstrate reconstitution of FGFR3 complexes *in vitro* for further mechanistic and structural studies. We also provide very comprehensive evidence (Figures 1 and S1–S4) that supports previously suggested correlation between thermal stability and the strength of client kinase recognition by Cdc37 (Taipale et al., 2012). Furthermore, we highlight the stability of an allosteric network within the N-lobe and the lobe interface as the main determinant of overall stability and consequently Cdc37 recognition (Figures 2, 3, and S3). We have shown that differences between weak and strong client kinases can be relatively subtle and involve the lobe interface as well as some less well-folded regions within the N-lobe (Figures 2 and 3). Nevertheless, a high degree of correct folding into a kinase structure appears to be essential for Cdc37 recognition (Figures 2 and 8).

We also show that recognition by Cdc37 is independent of the activation status, with one of the two highly oncogenic variants (N540K) being a very strong client, while the other (K650E) binds weakly with an affinity similar to the WT (Figure 1). This difference likely reflects distinct activation mechanisms: N540K substitution in the molecular brake (a feature that underpins intramolecular inhibitory constraints in the FGFR family within the N-lobe/lobe interface allosteric network) destabilizes an inactive conformation, thus facilitating activation, while the K650E variant introduces a substitution in the A-loop that stabilizes an active conformation by forming additional interactions (Chen et al., 2017, Huang et al., 2013). These findings also suggest that cancers driven by FGFR3 overexpression (as FGFR3 appears to be a better client compared with other FGFRs), or by FGFR3 mutations that further destabilize kinase structure, might be particularly worthwhile targets for exploration of Hsp90 and FGFR kinase inhibitor combinations as potential synergistic molecular therapies aimed at overcoming clinical drug resistance to kinase inhibitors as single agents.

The architecture of the kinase-cochaperone complex has not been defined for any of the client kinases, at any resolution. Based on HDX-MS applied to Cdc37, FGFR3 variants, and an unrelated sB-Raf^V600E^ kinase (Figures 4 and 6) in combination with the SAXS data for a FGFR3/Cdc37 complex (Figure 7), we show a relative orientation that could be adopted by various kinases in a binary complex. Interaction surfaces involve two areas, one formed by interaction between the C-terminal part of Cdc37 and the kinase C-lobe, and another between the N terminus of Cdc37 and a lobe interface/N-lobe of a kinase. This is consistent with previous studies suggesting a bipartite nature of Cdc37 binding to client kinases (Eckl et al., 2015, Keramisanou et al., 2016). Although sites of interactions on the kinase have not been directly analyzed in these studies, an assumption was made that the indispensable N terminus of Cdc37 interacts with the ATP-binding pocket at the lobe interface based on the Cdc37/kinase inhibitor or Cdc37/ATP competition (Eckl et al., 2015, Polier et al., 2013). Consistent with findings by Keramisanou et al. (2016), and a model for a two-step recognition, it is also possible that following initial interactions that involve the N terminus of Cdc37, features either pre-existing in strong clients or enhanced by Cdc37 in weaker clients could stabilize this area of interactions and allow for cooperative binding that involves distinct sites on Cdc37 (C terminus) and on the kinase (C-lobe).

Our data also suggest differences between Cdc37/kinase interactions in binary and ternary complexes. A recent cryoelectron microscopy structure of a Cdk4/Cdc37/Hsp90 ternary complex is consistent with multiple binding sites between the kinase and Cdc37, but does not include extensive involvement of the kinase C-lobe in these interactions; instead, a very localized interaction covering a short segment of the C-lobe at the lobe interface has been implicated (Verba et al., 2016). Because the surface area of Cdc37 that becomes protected in binary complexes is larger than if restricted to only kinase N-lobe and the lobe interface (Figures 6 and 7), it is conceivable that further rearrangements could be taking place in the ternary complex that also contains Hsp90. Furthermore, the separation of the N- and C-lobes of Cdk4 observed in the ternary complex, which involves formation of a linker consisting of unstructured β4 and β5 strands, is not supported by the analysis of the FGFR3/Cdc37 binary complex (either by HDX-MS, NMR, or CD), while our observation of more stringent kinase inhibition within the ternary complex suggests further changes caused by the chaperone (Figures 4, 5, and 8). In addition, the Cdk4/Cdc37 architecture seen within the ternary complex (Verba et al., 2016) would be energetically unfavorable without further stabilizing interactions of both proteins with Hsp90.

**Figure 9:**
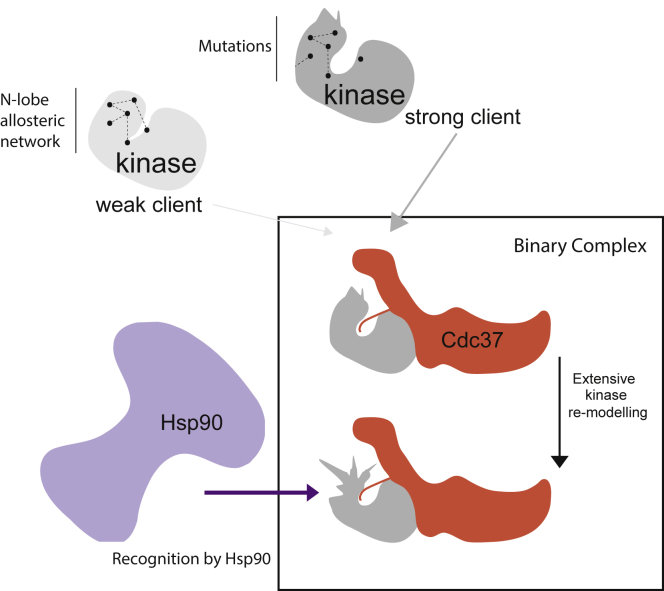
Insights into Formation, Architecture, and Functional Consequences of the Kinase/Cdc37 Complex Molecular determinants for differences between strong and weak clients arise from the degree of connectivity within the N-lobe allosteric network (top left). Interaction sites, identified on strong client kinases and Cdc37, mediate formation of stable binary complexes with defined structural organization. Interaction surfaces involve two areas, one formed by interaction between the C-terminal part of Cdc37 and the kinase C-lobe, and another between the N terminus of Cdc37 and a lobe interface/N-lobe of a kinase (center, top). Importantly, Cdc37 binding, via allostery, causes extensive remodeling affecting the entire N-lobe and results in kinase inactivation (center, bottom). Because the overall changes are substantial and the less compact, remodeled regions of the kinase left exposed in the binary complex, the main consequence of this re-modelling is to contribute to direct recognition by Hsp90 (bottom left). Within the ternary complex the kinase becomes further unfolded and interactions with, and relative orientation to, Cdc37 differ. Ultimately, the kinase is protected and refolded.

The mechanistic model proposed here (Figure 9) emphasizes extensive kinase structural and functional consequences of Cdc37 binding. These go beyond limited, localized changes that enhance the strength of binary client-cochaperone complexes, as proposed previously (Keramisanou et al., 2016). Unexpectedly, our data show that, in unrelated clients (FGFR3 variants and sB-Raf^V600E^), there is a similar, large conformational change upon Cdc37 binding, whereby the C-lobe shows a decrease in solvent exchange rate, indicating that it represents the direct binding site for Cdc37, while the N-lobe shows significantly increased solvent exchange rates (Figure 4). We argue that these increased rates of deuteration suggest remodeling with a degree of destabilization of the N-lobe and the impact of this effect is reflected in Cdc37's ability to inhibit kinase auto-phosphorylation (Figure 8). The phenomenon of N-lobe remodeling is further supported by our NMR data that show the Cdc37-induced emergence of a more flexible, transiently unfolded, N terminus (Figure 5). In our SAXS model, the kinase N-lobe is positioned to protrude (rather than be embedded) in the binary complex (Figure 7). Based on previous findings showing that Hsp90 interacts with the unfolded regions in its clients (Karagoz and Rudiger, 2015, Rohl et al., 2013), also supported here for FGFR3 (Figure 8), a likely implication is that the destabilized N-lobe provides a recognition site for the recruitment of and/or additional interactions with Hsp90 within a ternary complex. Moreover, this model of client destabilization to facilitate transfer to Hsp90 is also consistent with a well-supported model proposed for the glucocorticoid receptor (GR). Hsp70, which in this case facilitates client delivery to Hsp90, inactivates GR through partial unfolding (Kirschke et al., 2014). Cdc37 could have an analogous role for kinases to that of Hsp70 in the GR/Hsp70/Hsp90 system. Our mechanistic model (Figure 9) therefore provides a link between previous insights into structural domains of Cdc37 involved in entry of a client kinase into its chaperone cycle (Keramisanou et al., 2016) and the recent insight into the structural endpoint, namely a radically unfolded kinase within the ternary complex (Verba et al., 2016).

## STAR★Methods

### Key Resources Table

**Table:** 

REAGENT or RESOURCE	SOURCE	IDENTIFIER
**Antibodies**		

Mouse monoclonal anti-FGFR3	Abcam	Cat# ab204953
HRP-conjugated rabbit polyclonal anti FGFR3	LifeSpan Bioscience	Cat# LS-C214248
Rabbit polyclonal anti-FGFR1 phospho-Tyrosine 654	AbCam	Cat# ab70959; RRID:AB_1268885
Rabbit ployclonal anti-FGFR1 phospho-Tyrosine 766	AbCam	Cat# ab59180; RRID:AB_2293927
Goat anti-rabbit HRP-conjugate	GE Healthcare	Cat# NA934V

**Bacterial and Virus Strains**		

C41 (DE3)	Lucigen	Cat# 60442-1
XL-10 Gold	Stratagene	Cat# 200314

**Chemicals, Peptides, and Recombinant Proteins**		

Dovitinib	Stratech Scientific Ltd.	Cat# S1018-SEL
AZD4547	Stratech Scientific Ltd.	Cat# S2801-SEL
PD173074	Stratech Scientific Ltd.	Cat# 366905-USB
Rhamnose	Alfa Aesar	Cat# A16166
Spectinomycin	Apollo Scientific Ltd.	Cat# BIS0147
DNAse I	Sigma	Cat# D5025
Ulp1 protease	Expressed in-house	N/A
DMSO	Sigma	Cat# D2650
ATP	Thermo Fisher Scientific	Cat# R0441
SYPRO Orange Dye	Thermo Fisher Scientific	Cat# S6650
Pfu Ultra II	Agilent	Cat# 600670

**Deposited Data**		

FGFR3 backbone NMR assignment apo	BMRB	27082
FGFR3 backbone NMR assignment PD complex	BMRB	27083
SAXS Cdc37 model	https://www.sasbdb.org/	SASDBP9
SAXS Cdc37-FGFR3 I538F complex	https://www.sasbdb.org/	SASDBQ9
SAXS FGFR3 I538F	https://www.sasbdb.org/	SASDBR9

**Oligonucleotides**		

FGFR3^E466K^ mutagenesis fw GACCCCAAATGGAAGCTGTCTCGGGCC	This paper	N/A
FGFR3^E466K^ mutagenesis rv GGCCCGAGACAGCTTCCATTTGGGGTC	This paper	N/A
FGFR3^I538F^ mutagenesis fw GAAACACAAAAACTTCATCAACCTGCTG	This paper	N/A
FGFR3^I538F^ mutagenesis rv CAGCAGGTTGATGAAGTTTTTGTGTTTC	This paper	N/A
FGFR3^N540K^ mutagenesis fw CAAAAACATCATCAAACTGCTGGGCGCC	This paper	N/A
FGFR3^N540K^ mutagenesis rv GGCGCCCAGCAGTTTGATGATGTTTTTG	This paper	N/A
FGFR3^K650E^ mutagenesis fw CGACTACTACAAGGAGACAACCAACGGCC	This paper	N/A
FGFR3^K650E^ mutagenesis rv GGCCGTTGGTTGTCTCCTTGTAGTAGTCG	This paper	N/A

**Recombinant DNA**		

pOPINS FGFR3^WT^ Kinase Domain	(Bunney et al., 2015)	N/A
pOPINS FGFR3^E466K^Kinase Domain	This work	N/A
pJ821 FGFR3^I538F^ Kinase Domain	This work	N/A
pJ821 FGFR3^N540K^ Kinase Domain	This work	N/A
pJ821 FGFR3^K650E^ Kinase Domain	(Patani et al., 2016)	N/A
pJ821 sB-Raf^V600E^ Kinase Domain	This work	N/A
pET28 Cdc37	Gift from Cara Vaughan	N/A
pRSET Hsp90	Gift from Cara Vaughan	N/A
pTWO Casein Kinase II	Gift from Cara Vaughan	N/A

**Software and Algorithms**		

CCPNMR Analysis	(Vranken et al., 2005)	http://www.ccpn.ac.uk/v2-software/software/analysis
NMRPipe and NMRDraw	(Delaglio et al., 1995)	https://www.ibbr.umd.edu/nmrpipe/
ATSAS 2.8	(Franke et al., 2017)	https://www.embl-hamburg.de/biosaxs/software.html
Image Studio Lite	LiCor	https://www.licor.com/bio/products/software/image_studio_lite/
Mascot distiller	Matrix Science	http://www.matrixscience.com/distiller.html
HD-Examiner	Sierra Analytics	http://massspec.com/hdexaminer/
SigmaPlot	Systat Software Inc	www.sigmaplot.co.uk/products/sigmaplot/
CD Tools	(Lees et al., 2004)	N/A

**Other**		

Vanguard C18 pre-column	Waters	Cat# 186003975

### Contact for Reagent and Resource Sharing

Further information and requests for resources and reagents should be directed to and will be fulfilled by the Lead Contact, Tom D. Bunney (t.bunney@ucl.ac.uk).

### Experimental Model and Subject Details

For protein purification, *E. coli* C41 (DE3) transformed with either the Kan^R^ plasmid pOPINS or pJ821 and the Spec^R^ pCDF-Duet were plated on TB/Kan/Spec agar plates. Colonies were grown at 37° C in 500 ml TB medium or M9 minimal medium supplemented with 0.1 g/l kanamycin and 0.05 g/l spectinomycin until an OD_600nm_ of between 0.8 and 1.2 was reached. Cultures were then cooled to 15°C. Protein expression was induced by the addition of 0.1 mM IPTG (for pOPINS) or 1 mM rhamnose and 0.1 mM IPTG (for pJ821) to the cultures and incubation at 15° C for 16 h.

### Method Details

#### Cloning, Protein Expression & Site-Directed Mutagenesis

##### Preparation of Kinase Domains, Cdc37, Hsp90*α* and Casein Kinase II

*The FGFR3 kinase domain* (amino acids 455-768 of human FGFR3) was cloned into either pOPINS (OPPF, Oxford, UK) or pJ821 (DNA2.0, Menlo Park, USA) using In-Fusion cloning (Clontech, Mountain View, USA). Further procedures were similar as described in (Patani et al., 2016). Plasmids were transformed into C41 (DE3) cells harbouring a co-expression plasmid, pCDF-Duet, expressing lambda phosphatase under an IPTG inducible promoter. Transformed colonies were selected on agar plates containing Terrific Broth (TB), 10 mM Glucose, 100 μg/mL kanamycin and 50 μg/mL spectinomycin overnight at 37° C. Colonies were inoculated into liquid media containing the same components as the solid media and grown in baffled flasks to an optical density (OD^600^) of 0.8 to 1.2. Cultures were cooled to 15° C for 1 hour and then induced through the addition of 0.1 mM IPTG (for pOPINS) or 1 mM rhamnose and 0.1 mM IPTG (for pJ821) for 16 hours. Cells were pelleted and stored at -20° C until required. Mutations within all constructs were introduced using the site directed mutagenesis method (Agilent Technologies) and verified by sequencing of the complete open reading frame.

*The sB-Raf*^*V600E*^ (448-723 of human B-Raf) was purchased as a synthetic gene and cloned into pJ821 (resulting in a N-terminal His-SUMO tag). The protein incorporated mutations described in (Tsai et al., 2008). The expression was carried out identically as for FGFR3.

##### Cdc37, Hsp90*α* and Casein Kinase II

Expression plasmids for human Cdc37 (in pET28), Hsp90α (in pRSET) and Casein Kinase II (in pTWO), were kindly provided by C. Vaughan (Vaughan et al., 2006). Plasmids were transformed into C41 (DE3) cells and selected overnight on LB plates containing the appropriate antibiotic. Several colonies were inoculated into 0.5 L of 2xYT media containing appropriate antibiotics and grown to an OD_600_ of 0.4. Cultures were cooled to 25 °C for 2 hours and then expression was induced through the addition of 1 mM IPTG. Cultures were maintained overnight at 25 °C and harvested the following day by centrifugation and subsequent storage at -20 °C. For the production of Cdc37 with phosphorylated serine 13, the Cdc37 was co-expressed with the vector for CKII.

##### Expression of FGFR3 Kinase Domain Constructs in Minimal Medium (for ^15^N or ^15^N /^13^C Labelling)

Constructs were transformed as outlined above. Colonies were inoculated into 2 mL of TB containing 10 mM Glucose, 100 μg/mL kanamycin and 50 μg/mL spectinomycin and grown for 4 hours at 37 °C in an orbital shaker at 200 rpm. Minimal media agar plates were prepared containing 100 μg/mL kanamycin and 50 μg/mL spectinomycin with 3 plates necessary for every 500 mL planned culture. Each plate was spread with 100 μL of the TB culture and bacterial lawns allowed to grow overnight at 37 °C. Bacteria were scraped from the plates into 500 mL of minimal media containing a source of either ^15^N-ammonium sulphate (1g/L) alone or a combination of ^15^N-ammonium sulphate (1g/L) and ^13^C- glucose (5g/L). Cultures were grown in baffled 2 litre flasks for 1 hour at 37 °C and then 2 hours at 15 °C. The cultures were induced with the addition of 0.1 mM IPTG (for pOPINS) or 1 mM rhamnose and 0.1 mM IPTG (for pJ821) for 16 hours. Bacteria were pelleted and stored at -20 °C until processed.

##### Expression of FGFR3 Kinase Domain Constructs in Minimal Medium for Selective Unlabelling (for ^15^N Labelling with Particular Amino Acid Residues Unlabelled)

Constructs were transformed as outlined above. Colonies were inoculated into 2 mL of TB containing 10 mM Glucose, 100 μg/mL kanamycin and 50 μg/mL spectinomycin and grown for 4 hours at 37 °C in an orbital shaker at 200 rpm. Minimal media agar plates were prepared containing 100 μg/mL kanamycin and 50 μg/mL spectinomycin with 3 plates necessary for every 500 mL planned culture. Each plate was spread with 100 μL of the TB culture and bacterial lawns allowed to grow overnight at 37 °C. Bacteria were scraped from the plates into 500 mL of minimal media containing a source of ^15^N-ammonium sulphate (1g/L) and 1g/L of a particular amino acid or combination of amino acids. Cultures were grown in baffled 2 litre flasks for 1 hour at 37 °C and then 2 hours at 15 °C. The cultures were induced with the addition of 0.1 mM IPTG (for pOPINS) or 1 mM rhamnose and 0.1 mM IPTG (for pJ821) for 16 hours. Bacteria were pelleted and stored at -20 °C until processed.

##### Expression of FGFR3 Kinase Domain Constructs in Minimal Medium for Selective Labelling (for ^15^N Labelling with Only Particular Amino Acid Residues)

Constructs were transformed as outlined above. Colonies were inoculated into 2 mL of TB containing 10 mM Glucose, 100 μg/mL kanamycin and 50 μg/mL spectinomycin and grown for 4 hours at 37 °C in an orbital shaker at 200 rpm. Minimal media agar plates were prepared containing 100 μg/mL kanamycin and 50 μg/mL spectinomycin with 3 plates necessary for every 500 mL planned culture. Each plate was spread with 100 μL of the TB culture and bacterial lawns allowed to grow overnight at 37 °C. Bacteria were scraped from the plates into 500 mL of minimal media containing unlabelled ammonium sulphate and glucose and 1g/L of all amino acid (except tyrosine which was added at 0.5 g/L) and the amino acid to be labelled was not added at this point. Cultures were grown in baffled 2 litre flasks for 1 hour at 37 °C and then 2 hours at 15 °C. The labelled amino acid was added and then the cultures were induced with the addition of 0.1 mM IPTG (for pOPINS) or 1 mM rhamnose and 0.1 mM IPTG (for pJ821) for 16 hours. Bacteria were pelleted and stored at -20 °C until processed.

##### Expression of FGFR3 Kinase Domain Constructs in Deuterated Minimal Medium (for ^2^H/^15^N or ^2^H/^15^N /^13^C Labelling)

Constructs were transformed as outlined above. Colonies were inoculated into 2 mL of TB containing 10 mM Glucose, 100 μg/mL kanamycin and 50 μg/mL spectinomycin and grown for 4 hours at 37 °C in an orbital shaker at 200 rpm. Minimal media agar plates were prepared with deuterium oxide instead of water and contained 100 μg/mL kanamycin and 50 μg/mL spectinomycin with 3 plates necessary for every 500 mL planned culture. Each plate was spread with 100 μL of the TB culture and bacterial lawns allowed to grow for 48 hours at 37 °C. Bacteria were scraped from the plates into 500 mL of minimal media prepared with deuterium oxide and containing a source of either ^15^N-ammonium sulphate (1g/L) alone or a combination of ^15^N-ammonium sulphate (1g/L) and ^13^C- glucose (5g/L). Cultures were grown in baffled 2 litre flasks for 2 hours at 37 °C and then 4 hours at 15 °C. The cultures were induced with the addition of 0.1 mM IPTG (for pOPINS) or 1 mM rhamnose and 0.1 mM IPTG (for pJ821) for around 66 hours. Bacteria were pelleted and stored at -20 °C.

#### Protein Purification

Generally, pellets were retrieved from the -20 °C and resuspended in 20 mL of chilled Lysis Buffer (25 mM Tris.Cl, 250 mM NaCl, 40 mM Imidazole, 10 mM Benzamidine, 1 mM MgCl_2_ and 100 μM CaCl_2_, 100 μg/mL lysozyme, pH 8.0). Resuspension was accomplished by placing the pellets on an orbital shaker set at 200 rpm at 4 °C for 30 minutes. Lysis was continued by the addition of 5 mL of a solution of 10% (v/v) Triton-X-100 and 1 Kunit of bovine pancreatic DNAse I, on the orbital shaker at 200 rpm. at 4 °C for 1 hour. Clarification of the lysate was performed by centrifugation of the sample for 1 hour at 4 °C at 13,000 rpm in a JA-25.50 rotor (Beckman Coulter). All chromatography steps were performed on an Akta Explorer 10 (GE Healthcare, Amersham, UK) system.

##### Protein Purification Steps for Kinase Domains of FGFR1-4 and FGFR3 Variants

The clarified lysates were loaded onto a 5 mL HisTrap column (GE Healthcare, Amersham, UK). Proteins were washed with His Buffer A (25 mM Tris.Cl, 500 mM NaCl, 40 mM Imidazole, 1 mM TCEP, pH 8.0) and eluted with a 20-column volume gradient to His Buffer B (25 mM Tris.Cl, 500 mM NaCl, 500 mM Imidazole, 1 mM TCEP, pH 8.0). Eluted fractions containing protein were pooled and 100 μL of 10mg/mL Ulp1 protease was added per 20 mL of eluted recombinant protein. Removal of the purification tag progressed overnight in Dialysis Buffer (25 mM Tris.Cl, 1 mM TCEP, pH 8.0). Cleaved proteins were passed again over a HisTrap column using Dialysis Buffer as loading and wash buffer. Proteins that did not bind to the column were collected and subsequently injected on a 5 mL HiTrap Q (GE Healthcare, Amersham, UK) equilibrated in Q Buffer A (25 mM Tris.Cl, 20 mM NaCl, 1 mM TCEP, pH 8.0). Proteins were eluted over 20 column volumes to 50% of Q Buffer B (25 mM Tris.Cl, 1 M NaCl, 1 mM TCEP, pH 8.0) and eluted proteins collected in 10 mL fractions. Those fractions containing FGFR kinase domains were pooled and injected onto a Superdex 200 26/60 column (GE Healthcare, Amersham, UK) equilibrated in either NMR buffer (50 mM PIPES.NaOH, 50 mM NaCl, 2 mM TCEP, 1 mM EDTA, pH 7.0) or Standard Gel Filtration Buffer (25 mM Tris.Cl, 150 mM NaCl, 1 mM TCEP, pH 8.0). Monomeric FGFR3 kinase domain was concentrated in Vivaspin 10 kDa m.w.c.o. (Vivaproducts, Littleton, USA) concentrating units and quantified using a Nanodrop (Thermo Scientific, UK) using calculated molecular weight and extinction coefficients. Proteins were stored between 5 and 20 mg/mL, after snap freezing in liquid N_2_, at -80 °C.

##### Purification of HSP90*α*, CDC37^Apo^ (Non-Phosphorylated Cdc37) and CDC37^pSer13^ (Phosphorylated Cdc37)

The clarified lysates were loaded onto a 5 mL HisTrap column (GE Healthcare, Amersham, UK). Proteins were washed with His Buffer A (25 mM Tris.Cl, 500 mM NaCl, 40 mM Imidazole, 1 mM TCEP, pH 8.0) and eluted with a 20-column volume gradient to His Buffer B (25 mM Tris.Cl, 500 mM NaCl, 500 mM Imidazole, 1 mM TCEP, pH 8.0). Eluted fractions containing protein were dialysed overnight in Low Salt Buffer (25 mM Tris.Cl, 1 mM TCEP, pH 8.0). Proteins were subsequently injected onto a 5 mL HiTrap Q (GE Healthcare, Amersham, UK) equilibrated in Q Buffer A (25 mM Tris.Cl, 20 mM NaCl, 1 mM TCEP, pH 8.0). Proteins were eluted over 20 column volumes to 50% of Q Buffer B (25 mM Tris.Cl, 1 M NaCl, 1 mM TCEP, pH 8.0) and eluted proteins collected in 10 mL fractions. Those fractions containing the required protein were pooled and injected onto a Superdex 200 26/60 column (GE Healthcare, Amersham, UK) equilibrated in standard Gel Filtration Buffer (25 mM Tris.Cl, 150 mM NaCl, 1 mM TCEP, pH 8.0). Purification was monitored using SDS-PAGE and the relevant purified proteins were concentrated in Vivaspin 10 kDa m.w.c.o. (Vivaproducts, Littleton, USA) concentrating units and quantified using a Nanodrop (Thermo Scientific, UK) using calculated molecular weight and extinction coefficients. Proteins were stored between 5 and 20 mg/mL, after snap freezing in liquid N_2_, at -80 °C. Phosphorylation at serine 13 on CDC37 was verified using an antibody that specifically recognises this modification. This form was used in most experiments and designated as Cdc37 except when used to compare non- phosphorylated and phosphorylated forms.

##### sB-Raf^V600E^ Protein Purification

The clarified lysates were loaded onto a 5 mL HisTrap column (GE Healthcare, Amersham, UK). Proteins were washed with His Buffer A (25 mM Tris.Cl, 500 mM NaCl, 40 mM Imidazole, 1 mM TCEP, pH 8.0) and eluted with a 20-column volume gradient to His Buffer B (25 mM Tris.Cl, 500 mM NaCl, 500 mM Imidazole, 1 mM TCEP, pH 8.0). Eluted fractions containing protein were pooled and 100 μL of 10mg/mL Ulp1 protease was added per 20 mL of eluted recombinant protein. The protein was not dialysed. After 16 hours of cleavage the protein was desalted using four 5mL HiTrap Desalting columns (GE Healthcare) connected in series into Low Salt Buffer (25 mM Tris.Cl, 20 mM NaCl, 5%(v/v) glycerol and 1 mM TCEP, pH 7.0). The desalted mixture was then injected on a 5 mL HiTrap Q (GE Healthcare, Amersham, UK) equilibrated in B-Raf Q Buffer A (25 mM Tris.Cl, 20 mM NaCl, 5%(v/v) glycerol, 1 mM TCEP, pH 7.0). Proteins were eluted over 20 column volumes to 50% of B-Raf Q Buffer B (25 mM Tris.Cl, 1 M NaCl, 5%(v/v) glycerol, 1 mM TCEP, pH 7.0) and eluted proteins collected in 10 mL fractions. Those fractions containing B-Raf kinase domains were pooled and injected onto a Superdex 200 26/60 column (GE Healthcare, Amersham, UK) equilibrated in B-Raf Gel Filtration Buffer (25 mM Tris.Cl, 500 mM NaCl, 1 mM TCEP and 1 mM MgCl_2_, pH 7.0). Monomeric B-Raf kinase domain was concentrated in Vivaspin 10 kDa m.w.c.o. (Vivaproducts, Littleton, USA) concentrating units and quantified using a Nanodrop (Thermo Scientific, UK) using calculated molecular weight and extinction coefficients. Proteins were stored between 3 and 5 mg/mL, after snap freezing in liquid N_2_, at -80 °C.

#### Analysis of Protein Complexes

For analytical SEC, binary complexes were prepared with a kinase concentration of 15 μM and the Cdc37 concentration of 15 μM in a total volume of 40 μL. Ternary complexes were prepared similarly but included 30 μM of Hsp90α in addition to the kinase and Cdc37. Complexes were incubated at 4 °C for 15 min before loading onto an analytical Superdex 200 Increase column (24 mL, GE Healthcare) equilibrated in Gel Filtration Buffer (25 mM Tris.Cl, 150 mM NaCl, 1 mM TCEP, pH 8.0) at 4°C. Aliquots from 750 μL fractions were analyzed by SDS-PAGE and Coomassie staining; for samples containing FGFR3 kinase domain, aliquots were also used in ELISA (see below). For analysis of the effect of FGFR inhibitors on binary and ternary complex formation, the following compounds were used at 30 μM: Dovitinib, AZD4547 and PD173074 (all from Stratech Scientific Ltd.). In all cases the DMSO concentration was less than 0.15 %(v/v).

For analysis of binary complexes in PD experiments, 50 μg portions of Bait protein (Cdc37 or Hsp90 with HIS-tags) were immobilised on 100 μL of Talon resin (Clontech) in Wash Buffer (25 mM Tris.Cl, 150 mM NaCl, 1 mM TCEP, 0.1 %(v/v) Triton-X-100, pH 8.0) by incubation for 15 min at 4 °C on a rotating wheel. The Talon beads were subsequently washed three times with 500 μL of cold Wash Buffer to remove unbound protein. The target protein (FGFR3 kinase domain variants or *sB-Raf*^*V600E*^) (amounts specified in individual figures) was added to the immobilised Bait in 500 μL of Wash Buffer and incubated at 4 °C for 1 hour on a rotating wheel. The Talon beads were washed five times through the addition of 500 μL of cold Wash Buffer and gentle agitation of the beads followed by thorough aspiration. Proteins were eluted from the Talon beads through the addition of 50 μL Sample Buffer and 10 μL of 500 mM Imidazole. Proteins were subsequently denatured through a 5 min incubation at 95 °C and then loaded onto a 12% SDS-PAGE for separation and visualization by Coomassie staining or, when specified, by Western Blotting [FGFR3 specific antibody (LS-C214248, LifeSpan BioSciences)].

To quantify amounts of kinases present in binary and ternary complexes the gels were imaged using a Syngene Gel Documentation System and bands were quantified using Image Studio Lite (Licor). For comparison of FGFR3 variants, ELISA was also used to analyze individual samples and SEC fractions. ELISA plates (Immuno F96 Maxisorp, Nunc) were coated with a monoclonal (IgG) anti-FGFR3 antibody (55463, MP Biomedical) that had been diluted 1:8000 in Phosphate Buffered Saline (PBS) and incubated for 16 hours at 4 °C. The plate was washed once with ELISA Buffer (PBS containing 0.2%(v/v) Tween-20) and subsequently blocked using Sample/Antibody Diluent Buffer (PBS, 2%(w/v) BSA, 0.2%(v/v) Tween-20) and incubated for one hour at 25 °C in an orbital shaker. The plate was washed twice with ELISA Buffer. Several serial dilutions were prepared and 100 μL aliquots applied to the ELISA plate and incubated at room temperature for one hour. The ELISA plate was washed five times in ELISA buffer. One hundred μL of a 1:25,000 diluted (in ELISA Buffer) HRP-conjugated rabbit polyclonal anti FGFR3 antibody (LS-C214248, LifeSpan Bioscience) was added to each well and incubated for one hour at 25 °C in an orbital shaker. The ELISA plate was washed five times in ELISA buffer. The colourimetric substrate (555214, BD Bioscience) was added following manufacturer’s instructions and the plate read at 460 and 520 nm in a FLUOstar plate reader (BMG Labtech). Concentrations were interpolated from the standard curves using GraphPad Prism.

The data obtained by analyses of binary and ternary complexes were presented either as one representative image for two (or more) experiments, together with the quantitation of KDs corresponding to the image shown or, when stated in the figure legends, amounts of KDs were expressed as means +/- the standard deviation (SD) of three experiments.

#### Kinase Auto-Phosphorylation

All purified FGFR3 kinase domain proteins had been prepared in the presence of lambda phosphatase and were therefore in an unphosphorylated state. Aliquots of FGFR3 proteins (0.5 μg) were incubated in Kinase Buffer (25 mM Tris.Cl, 150 mM NaCl, 10 mM MgCl_2_, 1 mM MnCl_2_, 2 mM NaVO_4_, 0.1 mM TCEP, pH 8.0) and either CDC37^Apo^ (0-5 molar ratio) or Urea (0-5 M) for 30 min at room temperature. Subsequently, phosphorylation was initiated through the addition of 1 mM ATP and allowed to continue for 30 min at room temperature. Reactions were terminated through the addition of Sample Buffer and the proteins were denatured and separated on a 10 % SDS-PAGE. Proteins were identified through immunoblotting using antibodies recognising FGFR1 phosphorylated tyrosines 654 (ab70959, AbCam) and 766 (ab59180, AbCam) that presumably recognise FGFR3 phospho-tyrosines 648 and 760 respectively. Bands were detected with ECL Prime (GE Healthcare, Amersham, UK) and imaged digitally using a FujiFilm detector (FujiFilm Luminescent Image Analyzer, LAS-1000 CH). Bands were quantified using Image Studio Lite (LiCor). The data are shown as representative Western Blots and values obtained by image quantitation from three experiments plotted as means +/- the SD.

#### Thermofluor Assay

A PCR microplate was filled with 20 μL protein solution (1 mg mL^-1^ or 30 μM) of kinase variants in Thermofluor buffer (25 mM Tris.Cl, 150 mM NaCl, 1 mM TCEP, pH 8.0). Five μL of SYPRO Orange dye (ThermoFisher) was added to give a final dye dilution of 500 times the stock and a final sample volume of 25 μL. The microplates were sealed with an adhesive optical clear seal. Samples were heated using a RT-PCR instrument (iQ5, BioRad) and the increase in fluorescence monitored from 10 °C to 90 °C in 1 °C increments. The temperature at which 50 % of the protein is unfolded and bound to the fluorescent dye (apparent melting temperature, Tm) corresponds to the inflexion point of the slope. Raw data were plotted and analyzed using GraphPad Prism (http://www.graphpad.com). The transition midpoint was calculated automatically using an in-house written script. In experiments where inhibitors or nucleotides were added to the protein, a concentration of 60 μM was used. The final DMSO concentration in these experiments never exceeded 0.3 % (v/v). For each protein sample three to four aliquots were analyzed in the same experiment and data presented as the means +/- the SD.

#### ITC

Heats of interaction were measured on a VP-ITC system (Microcal) with a cell volume of 1.458 mL.

For titrations of FGFR and Cdc37, the FGFR3 kinase domain protein was dialyzed for 16 hours in ITC buffer (25 mM Tris.Cl, 50 mM NaCl, 5 mM MgCl_2_ and 0.1 mM TCEP, pH 7.5) at 4°C. FGFR3 ^WT^ or FGFR3^I538F^ variant were loaded in the sample cell (FGFR3^WT^ at 19.4 μM and FGFR3^I538F^ at 16.9 μM) and titrated with Cdc37^Apo^ in the syringe (211.7 μM). The titrations were performed while samples were being stirred at 260 revolutions per minute at 20 °C. A total of 20 injections were carried out, with 15 μL injected each time (except the first injection, when 3 μL was injected), and a 5 min interval between each injection to allow the baseline to stabilize. The data were fitted with a single site model to calculate the number of binding sites (n), the binding constant (Ka), the change in enthalpy (ΔH), and change in entropy (ΔS) using Origin software (Microcal, 2004).

#### CD

CD spectra were collected on a Jasco J-715 spectropolarimeter equipped with a temperature controller. The protein samples were prepared in 25 mM sodium phosphate, pH 7.5 with extensive dialysis. The CD Spectra were recorded at 20 °C with a step size of 1.0 nm, a bandwidth of 1 nm and an averaging time of 2.0 sec. Measurements were performed in a 1 mm path length quartz SUPRASIL cell (Hemlla, Germany) using 2.5 μM of proteins. Three scans were applied continuously and the data were averaged. The CD spectra were averaged, smoothed and processed after blank subtraction and converted to the presented units using CDtool software (Lees et al., 2004).

#### HDX-MS

Samples were prepared by incubating the proteins of interest at 5 μM either alone or in the presence of 7.5 μM binding partner on ice for 30 minutes in a buffer consisting of 25 mM Tris.Cl pH 8.0, 150 mM NaCl and 1 mM TCEP. Ten μL of each of these reactions was mixed rapidly with 40 μL of Deuterium Buffer (20 mM, Tris.Cl pH 8.0, 150 mM NaCl, 1 mM TCEP, 94.8%(v/v) D_2_O (Acros Organics), producing a final D_2_O concentration of 75.4 %(v/v). Four incubation conditions were performed in triplicate: three seconds on ice (which slows the exchange reaction by approximately tenfold, and is such representing a 0.3 second timepoint), three seconds at 22 °C, 30 seconds at 22 °C and 300 seconds at 22 °C. The reactions were quenched by the addition of 20 μL cold Quench buffer, consisting of 5 M Guanidinium Chloride and 8.4%(v/v) Formic Acid, followed by snap freezing in liquid nitrogen. Samples were then stored at -80°C.

The samples were sequentially thawed and injected onto an Ultra Performance Liquid Chromatography (UPLC) system submerged in ice. The sample was run over two in-series Poroszyme immobilised pepsin cartridges (Applied Bioscience) at a rate of 0.15 mL/min for three minutes, and the digested peptides were collected on a 1.7 μm Acquity UPLC BEH C18 VanGuard pre-column (Waters). The trap was then switched in-line to an Acquity UPLC BEH C18 column (Waters), and a 20 minute 5 to 45%(v/v) gradient of acetonitrile was passed over the column to elute the peptides, followed by a 4-minute wash of 76 %(v/v) acetonitrile. The mass of the eluted peptides was subsequently determined using a Waters Xevo quadrupole time of flight mass spectrometer. The instrument was acquiring for 30 minutes over mass/charge ratios of 350 to 1500, using an electrospray ionisation source operated at a capillary temperature of 225 °C and a spray voltage of 3.5 kV.

Peptides were identified from non-deuterated samples analysed using tandem mass spectrometry. A tolerance of 15 parts per million was used in the MS observations, and a tolerance of 0.2 Daltons in the MS/MS observations. The resulting datasets were analysed using Mascot Distiller (Matrix Science). Any peptide with a Mascot score ≤10 was excluded from further analysis. Centroid values were determined initially automatically using HD-Examiner software (Sierra Analytics), followed by manual quality control of each peptide. Because of the overlap from the shift in isotopic envelope observed upon deuterium incorporation, and peptides present from binding partners, additional peptides were removed from the dataset. All mentions of deuterium levels in the text and figures are relative values, as no fully deuterated sample could be obtained due to the buffer composition.

The data are expressed as means +/- the SD for triplicates at a given time point.

#### NMR

Uniformly ^15^N,^13^C,^2^H-labelled, uniformly ^15^N-labelled, selectively-labelled and selectively-unlabelled samples of FGFR3^WT^, free or in complex with non-labelled Cdc37, were prepared in 50 mM PIPES-NaOH, 50 mM NaCl, 5 mM TCEP and 1 mM EDTA (pH 7.0) containing 5% D_2_O. The GFR3^I538F^ variant, free and in complex with Cdc37, was prepared in an identical manner to that of GFR3^WT^, except that FGFR3^I538F^ singly labelled with ^15^N only was used for all experiments. All samples were approximately 200-300 μL and between 77 and 230 μM concentration in 5 mm Shigemi tubes. NMR spectra were recorded at temperatures ranging from 303K to 283K on Bruker Avance III or Avance III HD 600, 750, 800 or 950 MHz spectrometers equipped with 5mm QCI-P or TCI z-axis gradient Cryoprobes. Standard TROSY-detected triple-resonance experiments (Salzmann et al., 1998) and TROSY-detected HSQC experiments with water flip-back and WATERGATE pulses (Pervushin et al., 1998) were recorded as detailed previously. All data were processed using NMRPipe and NMRDraw (Delaglio et al., 1995) and analysed with CCPNMR Analysis (Vranken et al., 2005).

##### Assignments

^1^H-^15^N TROSY-HSQC spectra of uniformly ^15^N,^13^C,^2^H-labelled FGFR3^WT^, in free (230 μM) and PD173074-bound (1.30 equivalents of PD173074) forms were recorded at 303K as detailed previously (Bunney et al., 2015). The addition of PD173074 improved stability and spectral quality and was, therefore, used to facilitate assignment of free FGFR3^WT^. A 78% assignment of the non-Pro backbone amide resonances of the PD173074-bound FGFR3 HSQC spectrum was achieved using TROSY-based triple-resonance experiments (HNCO, HNCA, HN(CA)CB, HN(CO)CA, HN(CA)CO HNCB). Assignment of apo FGFR3^WT^ backbone amide and carbon resonances was accomplished by the use of TROSY triple-resonance experiments for backbone assignment (HNCA, HNCACB and HNCO). Samples of apo FGFR3 with selectively-labelled phenylalanine and leucine (U-^15^N-Phe, U-^15^N,^13^C-Leu) were used to solve ambiguities together with the assignment of PD173074-bound FGFR3 and by comparison to backbone assignments of free FGFR1 (Vajpai et al., 2014) and FGFR2 kinases (D. Cowburn and K. Dutta, personal communication). Ultimately, assignment of 75% of non-Pro backbone amides was achieved. Backbone assignments for PD173074-bound FGFR3^WT^ and apo FGFR3^WT^ have been deposited in the BioMagResBank database (accession numbers 27083 and 27082, respectively).

##### Titration with Cdc37

Uniformly ^15^N-labelled FGFR3^WT^ was titrated with non-labelled Cdc37 at various molar ratios (1:0.75, 1:1.9 and 1:3 FGFR3^WT^:Cdc37) and ^1^H-^15^N TROSY-HSQC spectra recorded for each titration point. Spectra of the FGFR3^WT^:Cdc37 complexes were recorded at 298 K and 283 K, the latter to improve sample stability. A series of FGFR3^WT^:Cdc37 complex spectra were also recorded at temperatures between these two extremes, and these used to track perturbation of backbone amide assignments due to temperature. The titration of FGFR3^I538F^ variant with Cdc37 was in a similar manner to FGFR3^WT^; ^1^H-^15^N TROSY-HSQC spectra were recorded for this variant in free and Cdc37 complex forms (1:0.25 and 1:0.50 molar ratios) at 283K. Transfer of backbone amide assignments from free FGFR3^WT^ to free FGFR3^I538F^ was completed where unambiguous, and CSPs calculated for those mutually assigned resonances, quantified as a combined chemical shift (Δδ) wherein Δδ = [(0.2^∗^δ^15^N_WT/I538F_)^2^+(δ^1^H_WT/I538F_)^2^]^0.5^. Comparison of ^1^H-^15^N TROSY-HSQC spectra of FGFR3^WT^:Cdc37 (1:0.70) and FGFR3^I538F^:Cdc37 (1:0.70) revealed the presence of sharp, random coil cross-peaks in the I538F complex spectrum at similar chemical shifts to those in the WT equivalent.

The identity of the new, slowly-exchanging amide cross-peaks was achieved using several approaches. Firstly, standard triple-resonance 3D TROSY-HNCA and TROSY-HN(CA)CB spectra were recorded on a ^15^N,^13^C,^2^H-labelled FGFR3^WT^:unlabelled Cdc37 complex sample to obtain connectivity and Cα/Cβ shift information to assist residue spin systems. Secondly, the HN and ^15^N random coil chemical shifts of FGFR3 backbone amides were predicted using a method described previously (Han et al., 2011) and compared to the experimentally-observed cross-peaks. Assignment was also facilitated using a selectively-unlabelled FGFR3:Cdc37 (1:1) complex composed of U-^15^N-FGFR3 with unlabelled Lys and Leu residues (-KL). All complexes were prepared using non-labelled Cdc37. Comparison of amide cross-peaks in the ^1^H-^15^N TROSY-HSQC spectra of the –KL selectively-unlabelled FGFR3:Cdc37 complex to those in a U-^15^N-labelled FGFR3:Cdc37 reference spectrum (1:0.75) was used to identify successfully unlabelled residues. All selectively unlabelled and reference spectra were recorded at 298K. To account for signal intensity variability due to sample concentration and stability, selectively unlabelled spectra were normalised with respect to the reference spectrum using G481, G533, G549 and G574 as reference peaks. Following normalisation, the peak height of each assigned cross-peak in the selectively unlabelled spectrum was divided by the intensity of the corresponding cross-peak in the reference spectrum to obtain an intensity ratio wherein 1 = equivalent intensities and 0 = completely unlabelled. To account for stochastic variation of peak height at different signal to noise ratios, peak height ratios for intensities at signal to noise ratios of 30, 20, 10 and 8 were simulated, and a confidence threshold corresponding to the mean peak height ratio minus 3 standard deviations was calculated for each. Backbone amide cross-peaks in the FGFR3:Cdc37 spectra were categorised with respect to their peak height signal:noise, then their peak height ratios compared to the appropriate confidence threshold. Only those cross-peaks with peak height ratios below their respective threshold are considered in the analysis.

#### SAXS

Synchrotron radiation small-angle X-ray scattering data were acquired for Cdc37, FGFR3^I538F^ variant and their complex at the P12 beamline of the European Molecular Biology Laboratory (EMBL) at the storage ring PETRA-III at the Deutsches Elektronen-Synchrotron (DESY), Hamburg. Using a Pilatus 2M detector at a sample-to-detector distance of 3.1 m and a wavelength of 0.124 nm, a momentum transfer range of 0.025< *s* < 5.0 nm^-1^ was covered (*s* = 4πsinθ/λ, where 2θ is the scattering angle). For each sample, SAXS data were acquired for a dilution series measured with the robotic sample changer (batch mode) and for an in-line size exclusion chromatography run (SEC-SAXS). Radial averaging and, for the batch mode data, frame averaging and buffer subtraction, were done using the SASFLOW within ATSAS 2.8 (Franke et al., 2017) analysis pipeline.

For Cdc37, the data from the batch mode dilution series (three concentrations ranging from 4.2 mg/mL to 1.1 mg/mL) were extrapolated to zero concentration using ALMERGE within ATSAS 2.8 (Franke et al., 2017). For FGFR3^I538F^ the scattering pattern was extracted from the SEC-SAXS intensity data (weighted for the estimated errors) using the non-negative matrix factorization within ATSAS 2.8 (Franke et al., 2017) (NMF) algorithm as implemented in the scikit-learn package within ATSAS 2.8 (Franke et al., 2017). The NMF fit was done with four components (one of which corresponds to the buffer), and the component with Guinier radius closest to that expected for FGFR3^I538F^ was selected for further analysis. This curve was then cropped using DATCROP within ATSAS 2.8 (Franke et al., 2017) to remove the data at the smallest angles affected by incorrect buffer suppression. For the FGFR3^I538F^:Cdc37 complex, the data from the batch mode dilution series (three concentrations ranging from 3.8 mg/mL to 1.0 mg/mL) were extrapolated to zero concentration using ALMERGE (Franke et al., 2017) then cropped using DATCROP (Franke et al., 2017) to remove the data at the smallest angles affected by interparticle interactions.

The forward scattering (I(0)) and the radii of gyration (*R*_g_) were evaluated using the Guinier approximation assuming that at very small angles, *s* < 1.3/*R*_g_, the intensity can be represented as $I\left(s\right)=I\left(0\right)\exp\left(\frac{{\left(sR_{g}\right)}^{2}}{3}\right)\text{.}$ These parameters were also computed from the entire scattering pattern using the indirect transform package GNOM within ATSAS 2.8 (Franke et al., 2017), which also provided the distance distribution function *P*(*r*) and the maximum dimensions of the particles (*D*_max_). The molecular mass (MM) of the solute was evaluated by comparison of the forward scattering with that from a reference solution of bovine serum albumin (MM = 66 kDa). Since the FGFR3^I538F^ data are extracted from a SEC-SAXS experiment, the forward scattering is not directly comparable with the reference solution and therefore no estimate of molecular mass is made. The particle volumes were computed using the Porod invariant (Svergun et al., 2013). Ambiguity analysis was performed with AMBIMETER within ATSAS 2.8 (Franke et al., 2017).

Two-phase *ab initio* models were reconstructed by MONSA using a dummy atom radius of 0.3 nm and a spherical search volume with diameter *D*_max_ = 16 nm (equal to the experimental maximum size of the complex) and P1 symmetry. The relative volumes of the individual phases were set proportionally to the number of amino acid residues in the respective proteins. The three SAXS curves for Cdc37, FGFR3^I538F^ and their complex were fitted simultaneously – the curves from individual proteins against the individual phases of the model, and the curve from the complex against the whole model. The MONSA calculations were repeated twenty times using different random seeds and the resulting models grouped into clusters using DAMCLUST within ATSAS 2.8 (Franke et al., 2017).

Rigid body modelling was carried out using CORAL within ATSAS 2.8 (Franke et al., 2017), using the available crystal structures of FGFR3 (PDB ID 4K33) and the C-terminal part of Cdc37 (PDB ID 1US7) together with the NMR structure of the N-terminal part of Cdc37 (PDB ID 2NCA, model A). Each of these three domains was free to move independently in the fit (constrained by the linker between 1US7 and 2NCA), but since the 4K33 and 1US7 structures have missing loops these were each modelled as two separate domains (before and after the loop) with fixed relative positions. The three SAXS curves were fitted simultaneously, with additional restraints using the interface information from the hydrogen-deuterium exchange mass spectrometry data. Specifically, the protected residues from FGFR3 kinase (Y607-L624, F667-F680, R728-L748) were requested to contact Cdc37. Reciprocally, the residues Y4-L28, R270-D293, F309-M316, L317-L341 from Cdc37 were requested to contact either FGFR3 or the other domain of Cdc37. The CORAL calculations were repeated twenty times using different random seeds and the resulting models grouped into clusters using DAMCLUST (Franke et al., 2017).

The experimental SAXS curves have been submitted to the Small Angle Scattering Biological Data Bank (SASBDB) with the accession codes; SASDBP9 for human Hsp90 co-chaperone Cdc37 protein (Cdc37), SASDBQ9 for human Hsp90 co-chaperone Cdc37 in complex with fibroblast growth factor receptor 3 kinase domain and SASDBR9 for human fibroblast growth factor receptor 3 kinase domain.

### Data and Software Availability

Backbone assignments for PD173074-bound FGFR3WT and apo FGFR3WT have been deposited in the BioMagResBank database (accession numbers 27083 and 27082, respectively). The experimental SAXS curves have been submitted to the Small Angle Scattering Biological Data Bank (SASBDB) with the accession codes; SASDBP9 for human Hsp90 co-chaperone Cdc37 protein (Cdc37), SASDBQ9 for human Hsp90 co-chaperone Cdc37 in complex with fibroblast growth factor receptor 3 kinase domain and SASDBR9 for human fibroblast growth factor receptor 3 kinase domain.

## Supplemental Information

Document S1. Figures S1–S7 and Table S2

Table S1. HDX-MS Source Data, Related to Figures 4 and 6 Relative percentage of global deuteration level of FGFR3WT (A), FGFR3 E466K (B), FGFR3 I538F (C), and B-Raf V600E (D) in the presence and absence of Cdc37. Relative percentage of global deuteration level of Cdc37 (E) in the presence and absence of FGFR3 E466K.

Document S2. Article plus Supplemental Information
