# Supplementary material for: Disease Variants of FGFR3 Reveal Molecular Basis for the Recognition and Additional Roles for Cdc37 in Hsp90 Chaperone System
Source: Structure. 2018 Mar 6;26(3):446–458.e8. doi: 10.1016/j.str.2018.01.016 (PMC5846801; doi:10.1016/j.str.2018.01.016)
Supplement: Document S1. Figures S1–S7 and Table S2 [file mmc1.pdf]

Structure, Volume *26*

Supplemental Information

Disease Variants of FGFR3 Reveal Molecular Basis for the Recognition and Additional Roles for Cdc37 in Hsp90 Chaperone System

Tom D. Bunney, Alison J. Inglis, Domenico Sanfelice, Brendan Farrell, Christopher J. Kerr, Gary S. Thompson, Glenn R. Masson, Nethaji Thiyagarajan, Dmitri I. Svergun, Roger L. Williams, Alexander L. Breeze, and Matilda Katan

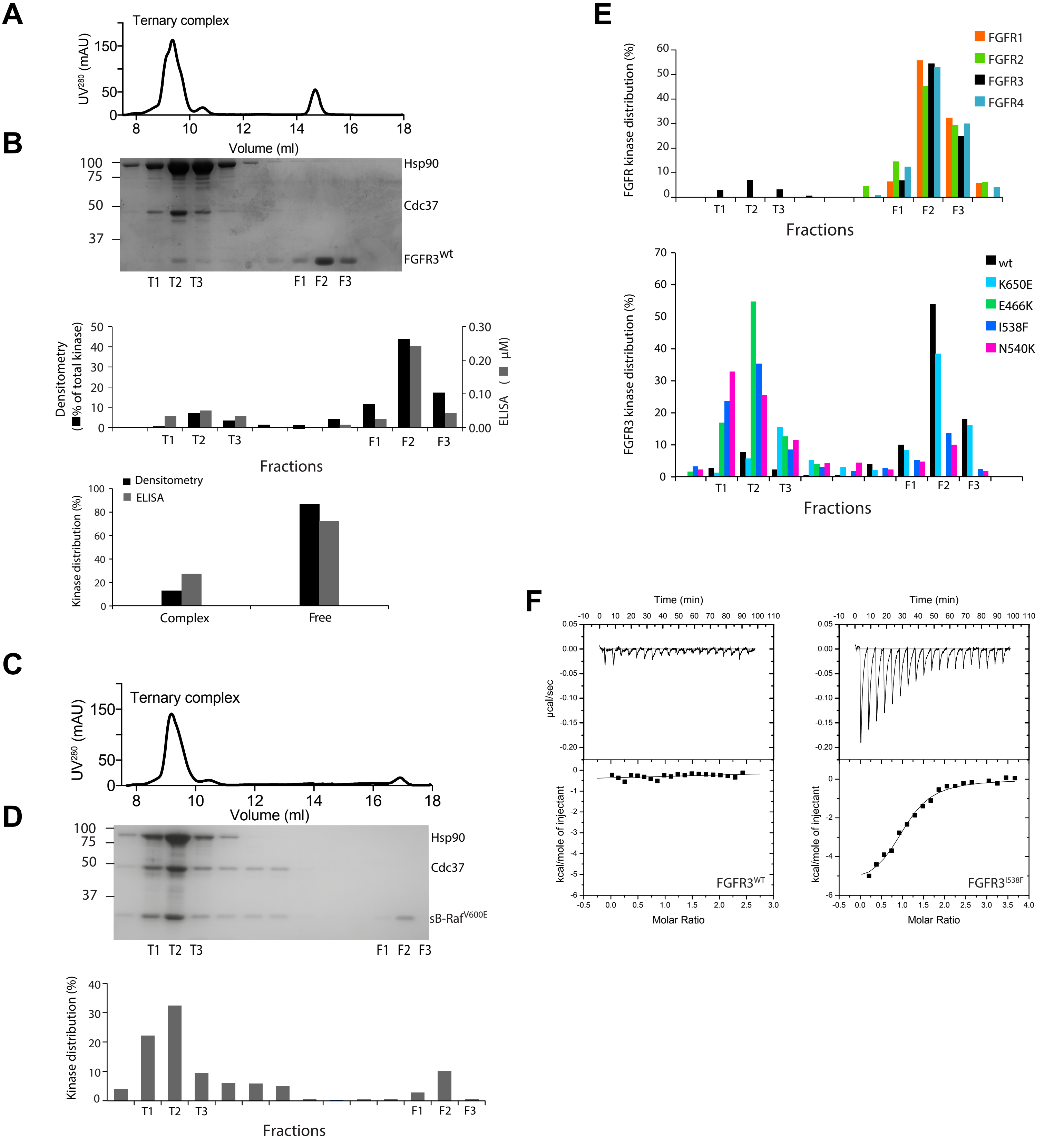

Figure S1 (Related to Figure 1). Quantitation of FGFR3 kinase domain interactions with Cdc37 and Cdc37/Hsp90 complexes. (A) SEC profile of the separation of a mixture (ratio Hsp90:Cdc37:FGFR3^{WT} of 2:1:1). (B) Coomassie stained gel of fractions taken from the SEC shown above showing the ternary complex (fractions T1-T3) and free FGFR3^{WT} (fractions F1-F3). FGFR3 kinase domain in individual fractions was quantified either using mAb in an ELISA assay or by densitometry of a Coomassie stained SDS-PAGE gel. (C) SEC profile of the separation of a mixture (ratio Hsp90:Cdc37: B-Raf^{V600E} of 2:1:1). (D) Coomassie stained gel of fractions taken from the SEC shown above showing the ternary complex (fractions T1-T3) and free B-Raf^{V600E} (fractions F1-F3). B-Raf^{V600E} in individual fractions was quantified by densitometry of a Coomassie stained SDS-PAGE gel. (E) The distribution of FGFR isoforms and FGFR3 variants between the ternary complex (T1 to T3) and free kinase (F1 to F3) determined by quantification of SEC data as outlined above. (F) (Left Panel) Heats generated upon injection of Cdc37 into a solution of FGFR3^{WT} at 20 °C. The interaction is too weak for analysis and the dissociation constant would be > mM. The experiment was repeated twice and one set of data is illustrated. (Right Panel) Heats of interaction upon injection of Cdc37 into a solution of FGFR3^{I538F} at 20 °C. The data is fitted with a 1 site binding model using Sigmaplot software with the ITC module. The following parameters were ascertained: The value of the dimensionless constant c was equal to 9.4. Stoichiometry (n) of 1.04 ± 0.04, Association constant (K_A) of 5.44 x 10^5 M^{-1} ± 9.81 x 10^4 M^{-1} (K_D of 1.8 μM), ΔH of -5.6 kcal mol^{-1} ± 0.3 kcal mol^{-1}, ΔS of 7.1 cal mol^{-1} K^{-1}. The experiment was repeated twice and one set of data is illustrated.

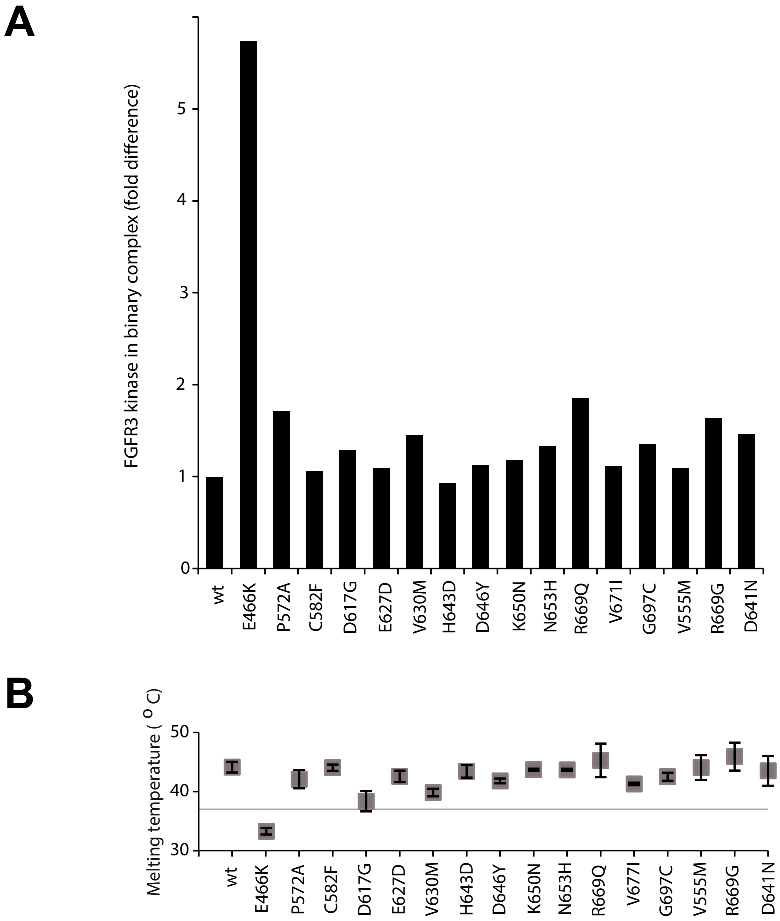

Figure S2 (Related to Figure 1). Relation between the binding affinity of FGFR3 variants for Cdc37 and their thermal stability. (A) Quantification of the amount of various FGFR3 variants binding to Cdc37 in binary complexes. Data is displayed as the fold difference in respect of $FGFR3^{WT}$. $FGFR3^{E466K}$ variant is included as a positive control. Representative experiment was repeated twice with similar results.

(B) Thermal stability of the same panel of mutants in (A). Line marks 37 °C. The SD errors are displayed for 3 replicates of each variant (n=3).

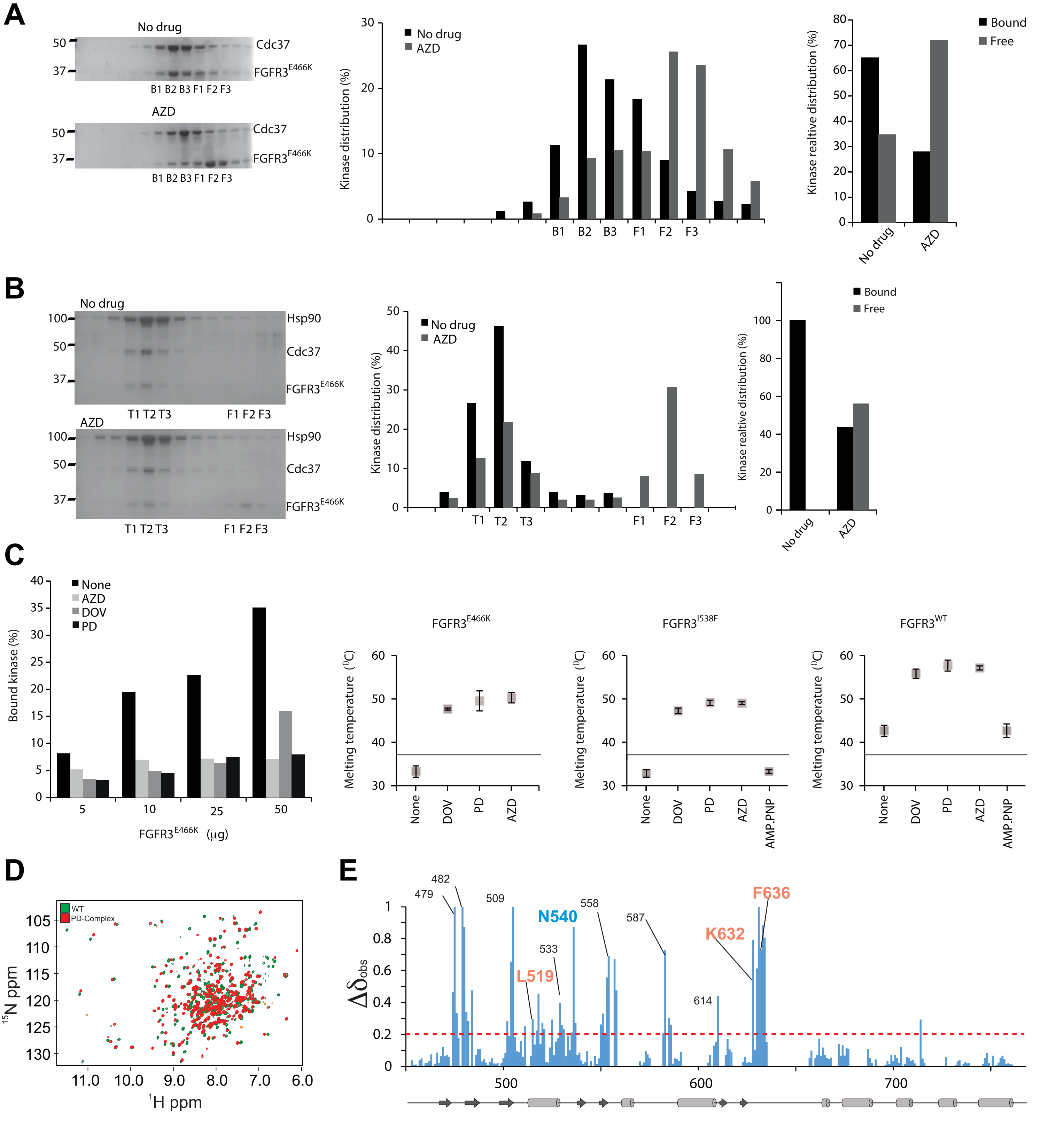

Figure S3 (Related to Figure 1). Effect of FGFR inhibitors on the formation of binary and ternary complexes. (A) SEC of binary complexes formed between FGFR3^{E466K} with Cdc37 in the absence (left panel) and presence (right panel) of the FGFR inhibitor AZD4547. Quantification of the kinase bands in the bound (B1 to B3) and free forms (F1 to F3) is illustrated in the lower panel left. A summary of the total bound and free kinase protein for the drug treated and untreated is shown in the far-right panel. Representative experiment repeated twice with similar results. (B) SEC of ternary complexes Hsp90:Cdc37:FGFR3^{E466K} formed without inhibitor (upper panel) and with AZD4547 (lower panel). Quantification of the kinase bands in the ternary complex (T1 to T3) and free forms (F1 to F3) is illustrated in the right panel. A summary of the total bound (ternary) and free kinase protein for the drug treated and untreated samples are shown in the far-right panel. Representative experiment repeated twice with similar results. (C) The effect of various FGFR inhibitors on the binding affinity of FGFR3^{E466K} to immobilised Cdc37 illustrated for various amounts of kinase (left panel). The effect of FGFR inhibitors and the non-hydrolysable ATP analogue (AMP.PNP) on the thermal stability of FGFR3 variants; the SD errors are displayed for 3 replicates of each condition (right panels). Representative experiment repeated twice with similar results. (D) Overlaid NMR spectra of FGFR3 in the apo state (green) and in complex with PD173074 (red). (E) CSP analysis of FGFR3 interaction with PD173074. Upon binding a substantial number of chemical shift perturbations are measured: labelled residues indicate the strongest shifts which are either part or proximal to the allosteric network identified in FGFR3 KDs.

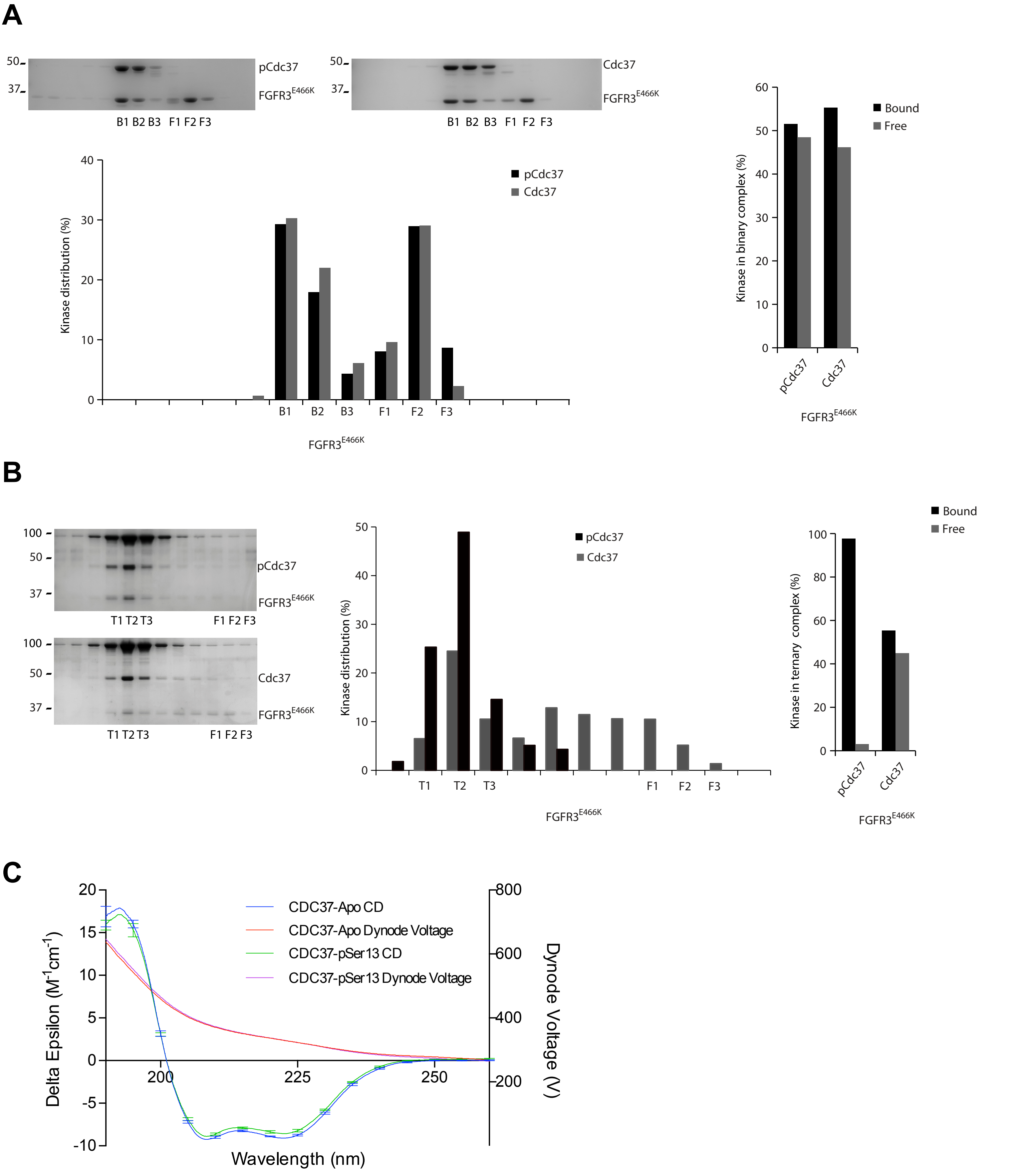

Figure S4 (Related to Figure 1). Effect of Cdc37 phosphorylation on the formation of binary and ternary complexes. (A) SEC showing binary complexes formed between $FGFR3^{E466K}$ with phosphorylated (pCdc37) (left panel) and non-phosphorylated Cdc37 (Cdc37) (right panel). Quantification of the kinase bands in the bound (B1 to B3) and free forms (F1 to F3) is illustrated in the lower panel. A summary of the total bound and free kinase protein for the non-phosphorylated and phosphorylated forms of Cdc37 is shown in the far-right panel. Representative experiment repeated twice with similar results. (B) SEC showing ternary complexes (Hsp90:Cdc37:$FGFR3^{E466K}$, 2:1:1) formed with pCdc37(upper panel) and Cdc37 (lower panel). Quantification of the kinase in the ternary complex (T1 to T3) and free forms (F1 to F3) is illustrated in the right panel. A summary of the total bound (ternary) and free kinase protein for the apo and phosphorylated forms of Cdc37 is shown in the far-right panel. Representative experiment repeated twice with similar results. (C) CD analysis of Apo Cdc37 and phosphorylated protein at serine 13. The data is shown between 190 and 260 nm and the dynode voltage is illustrated to show the accuracy of the analysis.

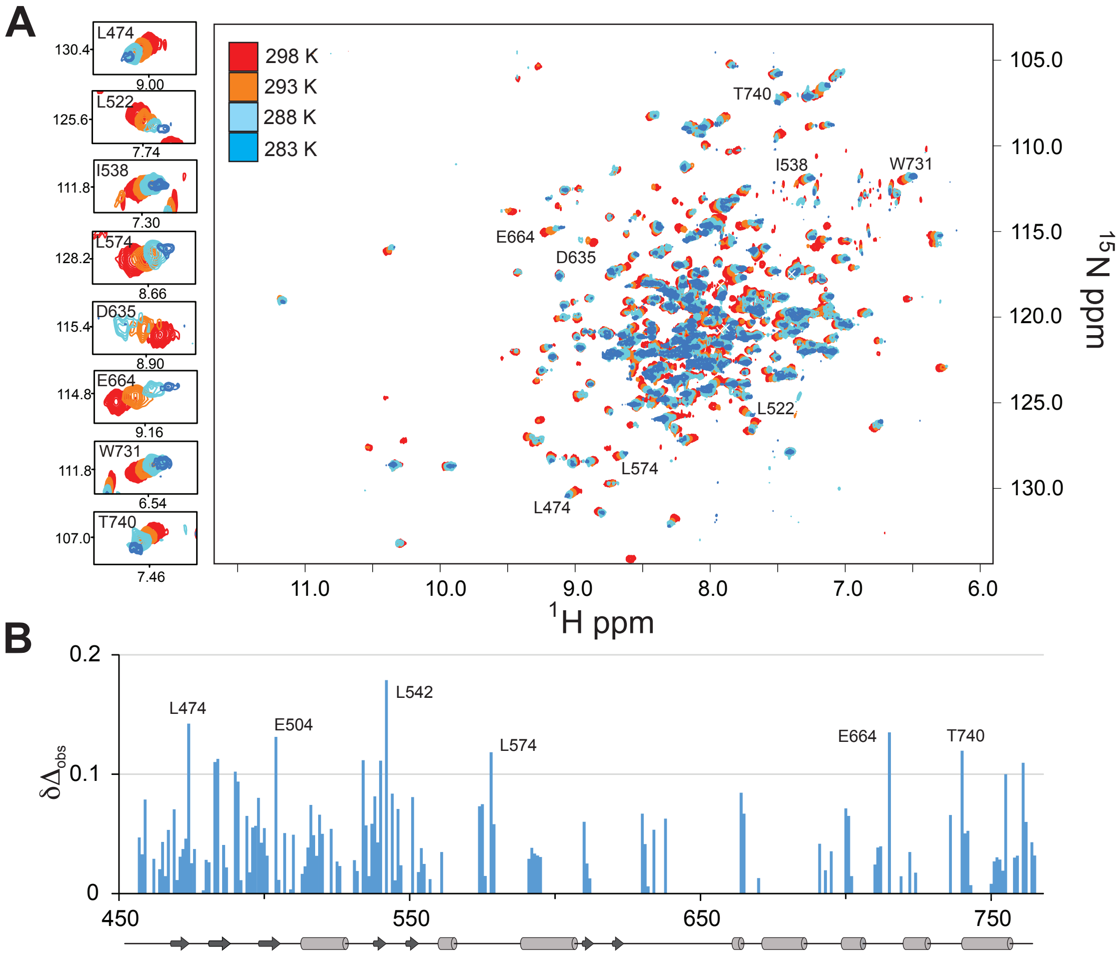

Figure S5 (Related to Figure 3). Temperature effects on FGFR3. (A) Overlaid 2D TROSY-HSQC of FGFR3^{WT} ranging from 298 K (red) to 283 K (blue). On the left are representative residues with significant chemical shifts. (B) Assessment of chemical shift perturbation. The histogram shows the quantitative analysis of the effects of temperature on FGFR3.

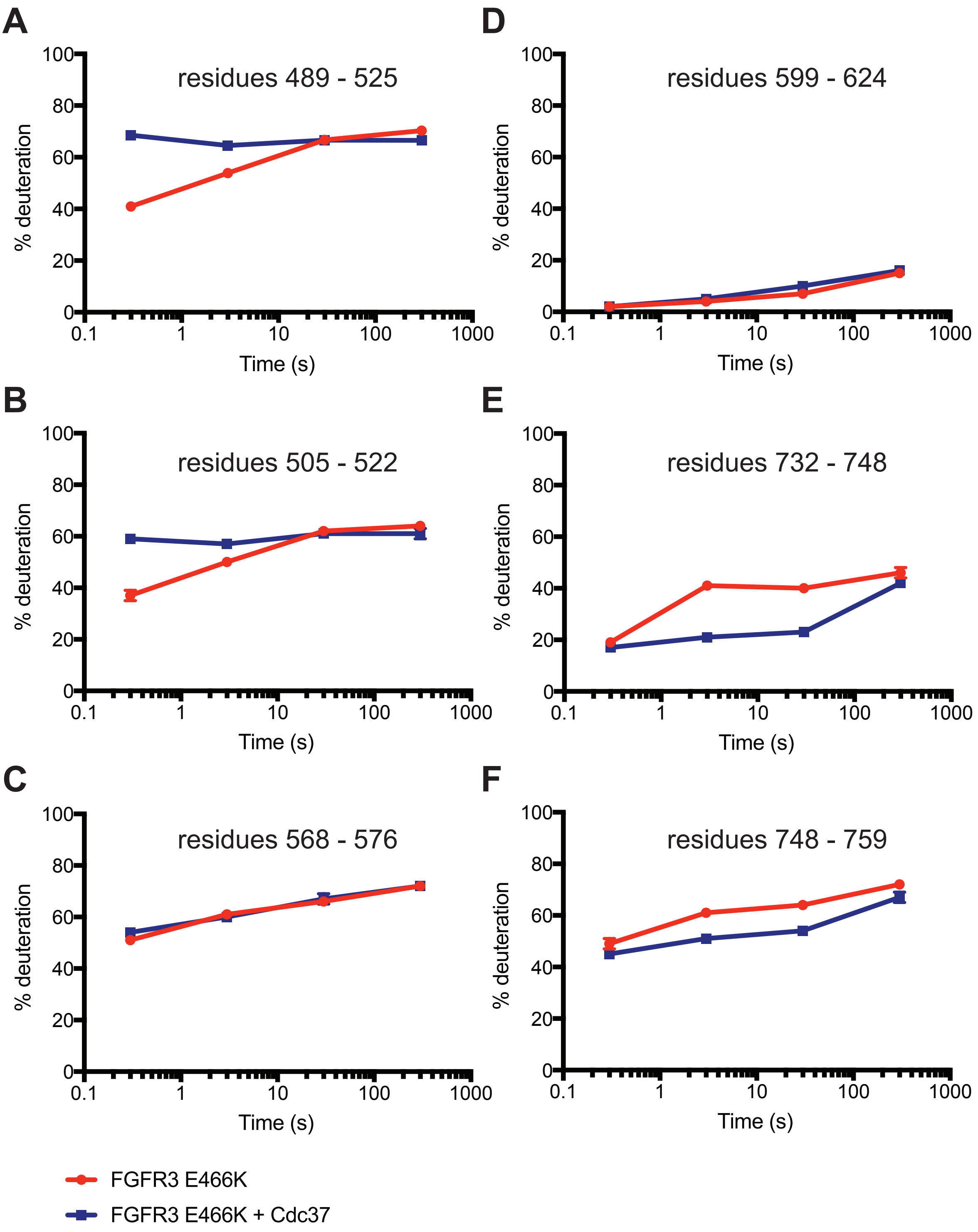

Figure S6 (Related to Figure 4). Uptake plots from six typical peptides across the FGFR3^{E466K} kinase domain. (A-F) show six typical plots indicating the percentage deuteration of individual peptides from a tryptic digest of the FGFR3^{E466K} kinase domain. Each peptide FGFR3^{E466K} from kinase domain complexed to Cdc37 is shown in blue or the equivalent peptide for FGFR3^{E466K} that is uncomplexed is shown in red.

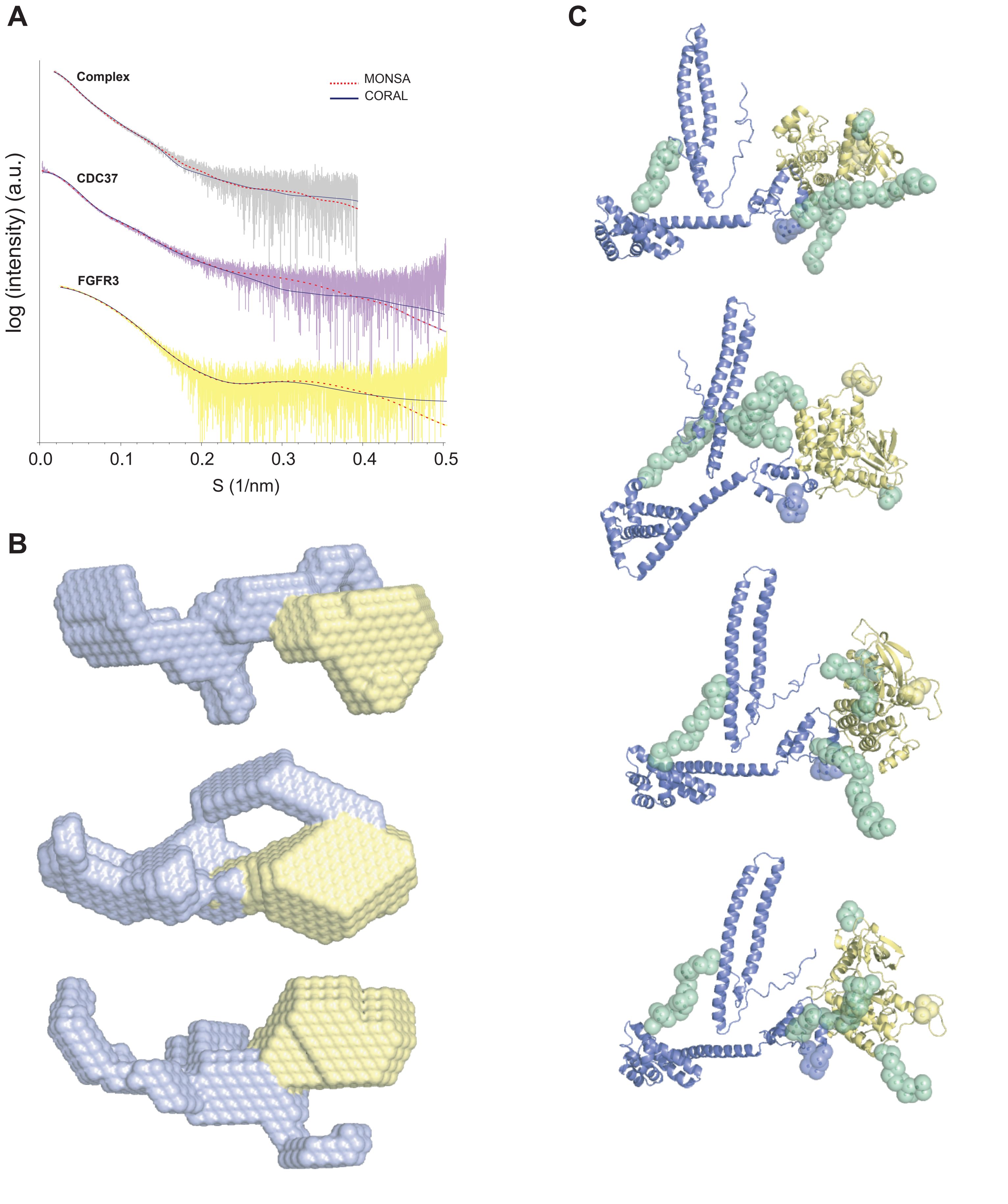

Figure S7 (Related to Figure 7). Generation of SAXS models. (A) Scattering curves for Cdc37 (Magenta), FGFR3^{I538F} (yellow) and the complex (grey). Overlaid to experimental data are the curves back-calculated from the models: dashed red is MONSA, blue CORAL. (B) Cluster of three representative dummy reconstruction obtained using MONSA. FGFR3^{I538F} is shown in yellow and Cdc37 in blue. (C) Cluster of four representative models obtained using CORAL. FGFR3^{I538F} is shown in yellow and Cdc37 in blue. Dummy atoms replacing missing protein portions are in light green.

Table S2 (related to Figure 7)

Data collection and model-free parameters.

	FGFR3^{I538F}	Cdc37	FGFR3-Cdc37 Complex
Data collection parameters			
Instrument	P12 at EMBL/DESY Storage Ring PETRA III		
Detector	Pilatus 2M		
Beam geometry	0.2 x 0.12 mm^2		
Wavelength (Å)	1.24		
q-range (Å^{-1})	0.008 – 0.47		
Exposure Time (ms)	20 x 45		
Concentration range (mg ml^{-1})	1.0 - 4.2		
Temperature (K)	293		
Structural parameters*			
Rg (from P (*r*)) (Å)	23.6 ± 0.2	41.9 ± 0.2	49.7 ± 0.4
Rg (from Guinier) (Å)	23.5 ± 2.0	40.0 ± 5.9	47.0 ± 2.0
Dmax	8.30 ± 3.0	15.53 ± 3.0	19.0 ± 5.0
Porod Volume (nm^3)	60 ± 5	106 ± 30	160 ± 20
Molecular mass determination*			
MM_{SAXS} (from *I*(0)) (kDa)	N/A	49 ± 5	77 ± 8
Calculated MM from Sequence (kDa)	35	44	80
SASBDB	SASDBR9	SASDBP9	SASDBQ9
Chi2 (MONSA)	1.2	1.6	1.4
Chi2 (CORAL)	2.8	1.9	2.2
Software employed			
Primary data reduction	Automated radial averaging		
Data processing	PRIMUS		
Ab initio analysis	DAMMIN, MONSA		
Validation and averaging	DAMAVER		
Rigid body modelling	CORAL		
Computation and model intensities	CRYSOL		

*Reported from infinite dilution of concentration series measurements
